# Long-read sequencing reveals the shell microbiome of apparently healthy American lobsters *Homarus americanus* from Atlantic Canada

**DOI:** 10.3389/fmicb.2023.1245818

**Published:** 2023-11-06

**Authors:** Svenja Koepper, K. Fraser Clark, J. Trenton McClure, Crawford W. Revie, Henrik Stryhn, Krishna K. Thakur

**Affiliations:** ^1^Department of Health Management, Atlantic Veterinary College, University of Prince Edward Island, Charlottetown, PE, Canada; ^2^Department of Animal Sciences and Aquaculture, Faculty of Agriculture, Dalhousie University, Bible Hill, NS, Canada; ^3^Department of Computer and Information Sciences, University of Strathclyde, Glasgow, United Kingdom

**Keywords:** microbial ecology, core microbiome, epizootic shell disease, 16S rRNA gene, PacBio, *Aquimarina*

## Abstract

The shell microbial community of lobsters—a key factor in the development of epizootic shell disease (ESD)—is still insufficiently researched in Atlantic Canada and many knowledge gaps remain. This study aimed to establish a baseline description and analysis of the shell microbiome of apparently healthy lobsters from four locations in the region. More than 180 lobster shell swab samples were collected from New Brunswick, Nova Scotia and Prince Edward Island (PEI). PacBio long-read 16S rDNA sequencing and bioinformatic analyses in QIIME2 identified the shell-associated bacteria. The shell microbiome of healthy lobsters consisted mainly of the bacterial classes *Gammaproteobacteria*, *Saprospiria*, *Verrucomicrobiae*, *Alphaproteobacteria*, *Flavobacteriia*, *Acidimicrobiia* and *Planctomycetia*. The microbial composition differed regionally and seasonally, with some classes showing decreased or increased relative abundances in the PEI samples as well as in the winter and spring samples in Nova Scotia. The core shell microbiome included potentially pathogenic as well as beneficial bacterial taxa, of which some were present only in certain regions. Bacterial taxa that have previously been associated with ESD were present on healthy lobsters in Atlantic Canada, but their frequency differed by location, sampling time, and moult stage. This study indicated that geographical and seasonal factors influenced the shell microbiome of apparently healthy lobsters more than host factors such as sex, size, and moult stage. Our results provide valuable reference microbial data from lobsters in a disease-free state.

## 1. Introduction

The American lobster (*Homarus americanus*) is an important species in the benthic ecosystem and supports Atlantic Canada’s most valuable fishery (DFO, 2021a). While lobster landings in Atlantic Canada are at an all-time high, lobster stocks in the southern range of the species have been threatened by ocean warming and disease outbreaks (Castro et al., 2012; Howell, 2012; DFO, 2021b).

Next-generation sequencing and faster computational methods have advanced microbial research in animals and humans over the past couple of decades. Multiple studies have highlighted the important influence of microbial communities on their host’s metabolism, growth, and immune functions (Berg et al., 2015; Bikel et al., 2015; Cornejo-Granados et al., 2017). Having a natural and healthy microbial community is thought to be beneficial for their hosts, as commensal bacteria can protect against invading pathogens, regulate the metabolism, synthesize vitamins, or help nutrient absorption (Ivanov et al., 2009; Venema, 2010; Bikel et al., 2015; Rajeev et al., 2021). The association between several chronic diseases and negative shifts in microbial communities—so-called dysbiosis—has changed the traditional view that a single pathogen is the cause of disease, towards a more holistic understanding where microbes and their host form one functional unit (Panda et al., 2014; Egan and Gardiner, 2016; Saraf et al., 2021).

Dysbiosis in the shell microbiome of American lobsters is hypothesized to be associated with the ecologically and economically important epizootic shell disease (ESD) (Meres et al., 2012). This syndrome is characterized by the formation of deep bacterial lesions on the carapace that spread rapidly over the whole lobster cuticle (Smolowitz et al., 2005; Cobb and Castro, 2006; Chistoserdov et al., 2012; Shields, 2013). Since its emergence in 1996, ESD has spread across most of the species’ range, most heavily affecting southern New England where lobster fisheries have decreased drastically since the emergence of ESD (Glenn and Pugh, 2006; Castro and Somers, 2012; Groner et al., 2018). While some bacterial species have been associated with ESD lesions (e.g., *Aquimarina*, *Thalassobius*) it is not clear whether these are causative agents or simply secondary invaders (Chistoserdov et al., 2012; Meres et al., 2012; Quinn et al., 2013, 2017). To determine ESD risk factors and why certain regions and lobster demographics are more susceptible to this disease, a general understanding of the lobster shell microbial community is necessary (Egan and Gardiner, 2016).

Previous research has identified the classes *Acidimicrobiia*, *Alphaproteobacteria*, *Bacteroidota*, *Myxococcota* (formerly: *Deltaproteobacteria*), *Flavobacteriia*, and *Gammaproteobacteria* as common members of the shell microbial community in lobsters unaffected by ESD. However, many former studies were restricted to small sample sizes often taken from lab-reared animals or did not use next-generation sequencing. Consequently, with no comprehensive studies of wild and healthy American lobsters, we lack comparable baseline data and are unable to determine which factors or microbial taxa impact the shell microbiome during disease onset and proliferation.

This study aimed to (1) describe the shell microbiome of apparently healthy lobsters sampled from four locations in Atlantic Canada, (2) determine which bacterial taxa make up the core shell microbiome and whether these differ by geographical, seasonal or host factors, and (3) ascertain whether ESD-associated taxa, in particular the genus *Aquimarina*, are present in the shell microbiome and, if so, whether there are risk factors for their frequency. As members of the *Aquimarina* genus have been linked to ESD in lobsters (Chistoserdov et al., 2012; Meres et al., 2012), it was also of interest how this genus was distributed in this study. Using third-generation long-read sequencing, we present a comprehensive description of the shell microbiome of lobsters from historically ESD-free regions. This dataset offers valuable baseline information on, what we define as, the “healthy” shell microbiome of lobsters in Atlantic Canada and the factors influencing it. It enhances our understanding of what roles certain microbes might play in the healthy lobster shell microbiome and how this may affect the development of ESD.

## 2. Materials and methods

### 2.1. Sample collection

**Figure 1:**
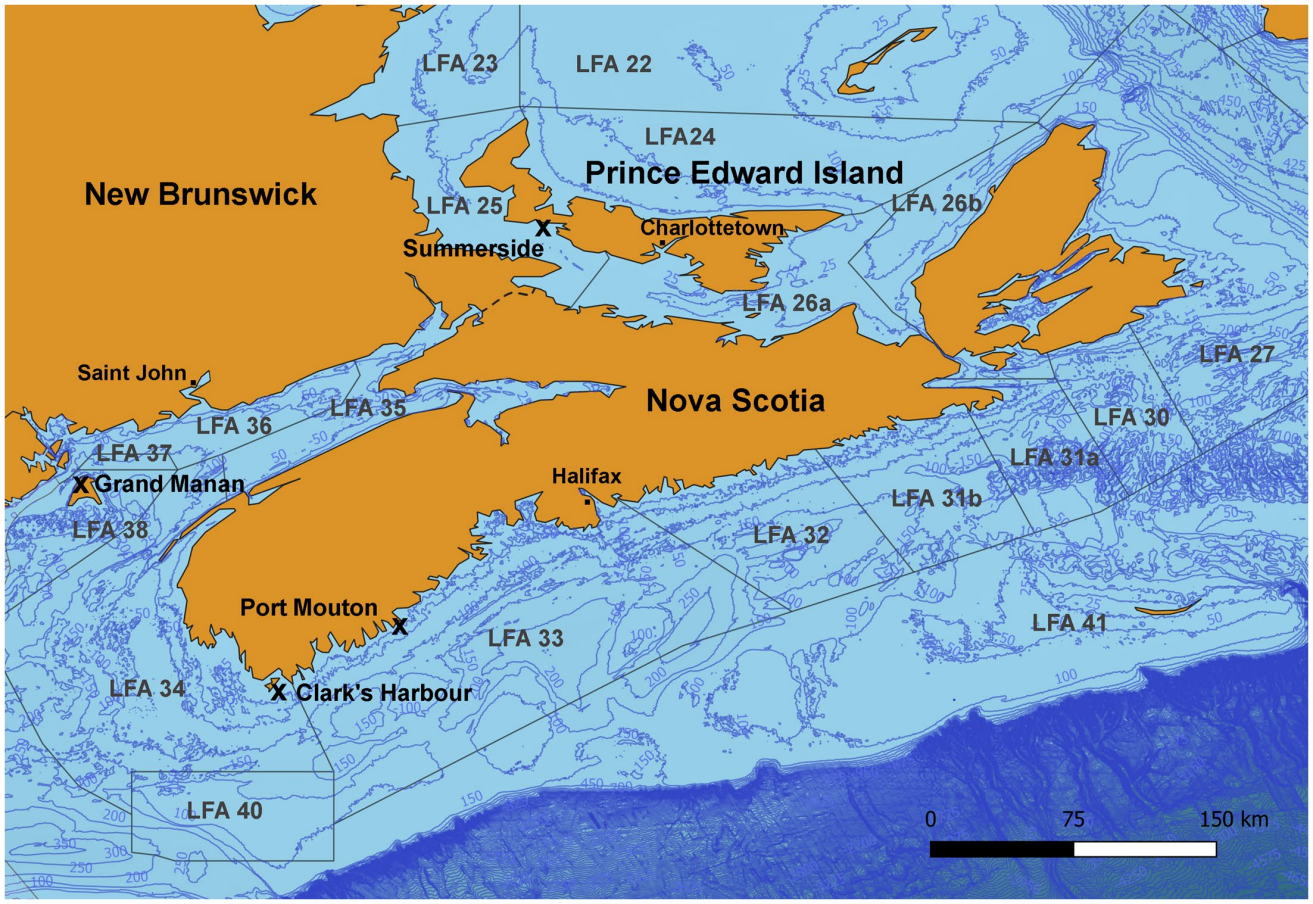
Map of the sampling areas in three Atlantic Canadian provinces and landing ports are indicated by an “x.” In Port Mouton (LFA33) and Clark’s Harbour (LFA 34), sampling occurred twice, once in December 2021 and again in May 2022. In Summerside (LFA 25), sampling occurred twice in September 2021 and October 2022).

Shell microbiome samples from freshly landed lobsters were collected from commercial fishing boats; once in New Brunswick [Grand Manan, Lobster Fishing Area (LFA) 37] and twice, respectively, in Nova Scotia (Clark’s Harbour, LFA 34; Port Mouton, LFA 33) and Prince Edward Island (Summerside, LFA 25) (Table 1 and Figure 1). The preferred way of sampling was to swab freshly trapped lobsters directly on fishing vessels. In cases where this was not possible, e.g., due to weather, samples were taken from freshly landed lobsters at the wharf. To determine any effect of these sampling methods on the shell microbiome, a direct comparison between boat and wharf sampling was implemented in Summerside (LFA 25) where during one sampling event (October 2022) lobsters were sampled on the boat directly after coming out of the water and again, at the wharf, after being in lobster crates for 6 h. Lobsters that showed signs of ESD were swabbed twice, once on shell disease lesions and once on an apparently healthy part of the carapace.

**Table 1:** Descriptive and summary statistics of sampled apparently healthy lobsters (*N* = 189).

Province	LFA	Location	Date	*N*	Site	Median size (Q1, Q3)	Moult stage (I/P)	Median depth (Q1, Q3)	Sex (M/F/B)
PEI	25	Summerside	September 2021	24	B	75 (73, 81)	(9/15)	14.3 (14.3, 16.7)	(11/13/0)
			October 2022	24	B	86 (78.5, 90.5)	(20/4)	19.7 (18.1, 20.4)	(14/8/2)
			October 2022	19	W	89 (84, 90)	(15/4)	NA	(11/8/0)
Nova Scotia	33	Port Mouton	December 2021	28	B	94.5 (88, 104.5)	(27/1)	28.0 (25.6, 29.3)	(19/7/2)
			May 2022	23	B	86 (81, 92)	(23/0)	10.4 (8.6, 11.9)	(11/10/2)
	34	Clark’s Harbour	December 2021^*^	23	W	88 (84, 93)	(21/2)	NA	(17/6/0)
			May 2022	24	B	81 (77.5, 93)	(24/0)	17.2 (9.5, 23.2)	(17/7/0)
New Brunswick	37	Grand Manan	May 2022	24	W	98 (89.5, 104.5)	(24/0)	NA	(13/10/1)

Median size (carapace length, in mm) and median water depth (in m) with 25th (Q1) and 75th (Q3) percentiles. B, boat; W, wharf; I, intermoult; P, postmoult; M, male; F, female; BF, berried female.

Samples were taken as described in Koepper et al. (2023). Briefly, sterile cotton-tipped applicators were used to collect the lobster shell microbiome. The left side of the carapace was washed with 5 mL of sterile water (DNase/RNase/protein-free) filtered through a 0.22 μm filter mounted on a 60 mL syringe. Then the cotton swab was dipped in aliquoted Buffer A (20 mM Tris-HCl, 2 mM sodium EDTA, 1.2% Triton X-100, pH 8.0) (Birer et al., 2017) and used to swab the washed carapace region. To collect seawater controls, sterile cotton swabs were submerged in seawater at the respective sampling locations. Swabs were stored in 100% ethanol at room temperature until further processing.

### 2.2. Bacterial 16S DNA extraction and sequencing

Bacterial DNA was extracted using the Qiagen Blood and Tissue kit with an additional prior lysis step: swabs were placed in new 1.5 mL microcentrifuge tubes and incubated at 56°C until all ethanol evaporated. Then 300 μL of lysozyme reaction mix (1:10 lysozyme (11 mg/mL): lysozyme buffer (1 M NaCl, 0.5 M EDTA pH 8.0, 1 M Tris-HCL pH 8.0, sterile water) were added to each sample followed by a 30 min incubation at 37°C. The next steps followed the kit manufacturer’s instructions (starting at step 1a). Several extraction controls of molecular biology grade water were added right before sequencing. The quantity and quality of extracted DNA of the samples were checked by Nanodrop (Thermo Scientific, NanoDrop Spectrophotometer ND-1000). Extracted DNA was shipped to the Integrated Microbiome Resource Lab at Dalhousie University, Halifax, Nova Scotia for full-length 16S rRNA gene PacBio sequencing, where library preparation and subsequent sequencing took place according to their in-house protocols (Comeau et al., 2017a,b). In summary, the whole 16S rDNA region was amplified once prior to sequencing (40 cycles) with the full-length fusion primers 27F [AGRGTTYGATYMTGGCTCAG, (Paliy et al., 2009)], 1492R [RGYTACCTTGTTACGACTT, (Lane, 1991)] specific to the PacBio Sequel 2 system and PCR products were validated using a Coastal Genomics Analytical Gel. The PCR reactions were then cleaned and normalized using Charm Biotech Just-a-Plate 96-well Normalization Kit. Samples were pooled on plates (separate wells) and fluorometrically quantified to make one library. Lastly, single-molecule-real-time (SMRT) sequencing was performed by the PacBio Sequel 2 platform on 8 M chips with 240–320 Gb output (Comeau et al., 2017a,b).

### 2.3. Bioinformatic analysis

All bioinformatic steps were performed using the open-source bioinformatics platform QIIME2 via the Galaxy interface run through Docker on Windows (Bolyen et al., 2019; q2galaxy, 2023). The sequencing produced demultiplexed FASTQ files, which were processed in QIIME2 according to the PacBio CCS Amplicon standard operating procedure (Version 1) (Comeau et al., 2017b) after inspecting the read quality of the raw sequence. Briefly, forward and reverse primer sequences were trimmed with “cutadapt” (forward: 1,200, reverse: 18,000) and FASTQ read files were imported into QIIME2 via a manifest file. The DADA2 algorithm was used to denoise the reads and create amplicon sequence variants (ASVs) (Callahan et al., 2016). ASVs that appeared in only a single sample were removed. Taxonomy was assigned with a Bayesian-trained classifier (Greengenes 13_8 99%, McDonald et al., 2011) and ASVs of mitochondrial and eukaryotic origin were removed from the dataset. Although the seawater and extraction control samples were sequenced along with the swab samples, they failed the PacBio sequencing due to low DNA content and were subsequently removed during quality filtering.

### 2.4. Statistical analyses

To explore the microbial composition on several taxonomic levels, ASV frequencies were plotted on the phylum level (heat map), relative abundances on class level (bar graph) and the core microbiome and the frequencies of ESD-associated taxa were analyzed on the lowest possible taxonomic level. The relative abundances were calculated by merging ASVs by the respective taxonomic level and then dividing the abundance (number of reads) of one taxon by the total abundance of all taxa in a sample. The relative abundances per sample were subsequently plotted as a bar graph for the nine most abundant bacterial classes. The ASV phylum counts were Hellinger transformed by square rooting the relative abundances to plot the right skewed ASV frequencies in a heatmap. The web-based visualization and analysis tool Clustergrammer was used to create heatmaps (Fernandez et al., 2017).

The core microbiome was determined with the QIIME2 plugin “core-features” for each comparison group separately, e.g., for samples from male, female, and berried lobsters. Core taxa were defined as being present in at least 80% of the samples. The Venn diagrams presenting the amount of shared and unique core microbiome ASVs among sampling groups were created using the interactive web tool Venny (Oliveros, 2007).

To determine whether the frequencies of ESD-associated taxa were associated with any geographical, seasonal or host factors (sex, size, moult stage), initial descriptive statistics (occurrence, prevalence, mean and total number of reads, minimum and maximum counts of detected bacterial taxa) were summarized for the each of the previously identified ESD-associated taxa, according to literature [*Aquimarina, Flavobacterium, Kiloniella, Polaribacter, Pseudoalteromonas, Sulfitobacter*, *Thalassobius* and *Vibrio*; (Chistoserdov et al., 2012; Meres et al., 2012; Feinmann et al., 2017; Ishaq et al., 2023)]. The frequencies of these taxa were investigated, to assess their association with geographical, seasonal or host factors by building a regression model for count data. In initially fitted Poisson models, the variances of the bacterial frequencies were larger than the mean, suggesting overdispersion. Accordingly, the negative binomial model type with the best fit for the data was the NB1 negative binomial which assumes a constant mean. Subsequently, negative binomial regression (NB1) models were built for each of the taxa with the respective taxon frequency as the outcome and using LFA, sampling month, lobster sex, size and moult stage as explanatory variables. The models did not include an offset since there was no population at risk present. This analysis was conducted in Stata (Release 17, StatCorp, College Station, TX, United States, 2021). Forward model building was used, where variables that were associated with the outcome (bacterial taxon count) with a *p*-value <0.05 were kept in the multivariable final model. A *p*-value of 0.05 was considered significant for fixed effects and interactions. To gain a better understanding of the *Aquimarina* strains present on healthy lobster shells, respective ASVs of this genus were also assigned by the blast algorithm (Altschul et al., 1990). The above-described analysis of ESD-associated taxa was also performed on the rarefied dataset (normalized to 400 reads per sample, *n* = 185 samples). This was done as a sensitivity analysis to evaluate the use of rarefaction for these types of microbiome analysis.

## 3. Results

### 3.1. Descriptive statistics

Overall, the shell microbiome of 189 lobsters (19–28 lobsters per sampling event) was analyzed, of which 113 were male, 69 were female and 7 were egg-bearing females. Only one ESD-diseased lobster was sampled (LFA 33 in December), while all others were apparently healthy and showed no signs of shell disease. Lobster sizes ranged from 55 mm CL to 129 mm CL (median: 87 mm, Q1: 81 mm, Q3: 96 mm). Sampled male lobsters were slightly larger than female lobsters with a median of 88 mm CL compared to 87 mm CL. Berried female lobsters were the largest with 92 mm CL (median). 86% of lobsters (163 animals) were in the intermoult stage, whereas 14% (26 animals) were in postmoult stage. For samples collected on commercial fishing vessels, the fishing depth ranged from 7.5 m to 36.6 m (median: 15.2 m, Q1: 13.7, Q3: 22.0 m). For samples taken from the wharf, no water depth data was available. The summary statistics for each sampling event are shown in Table 1.

**Figure 2:**
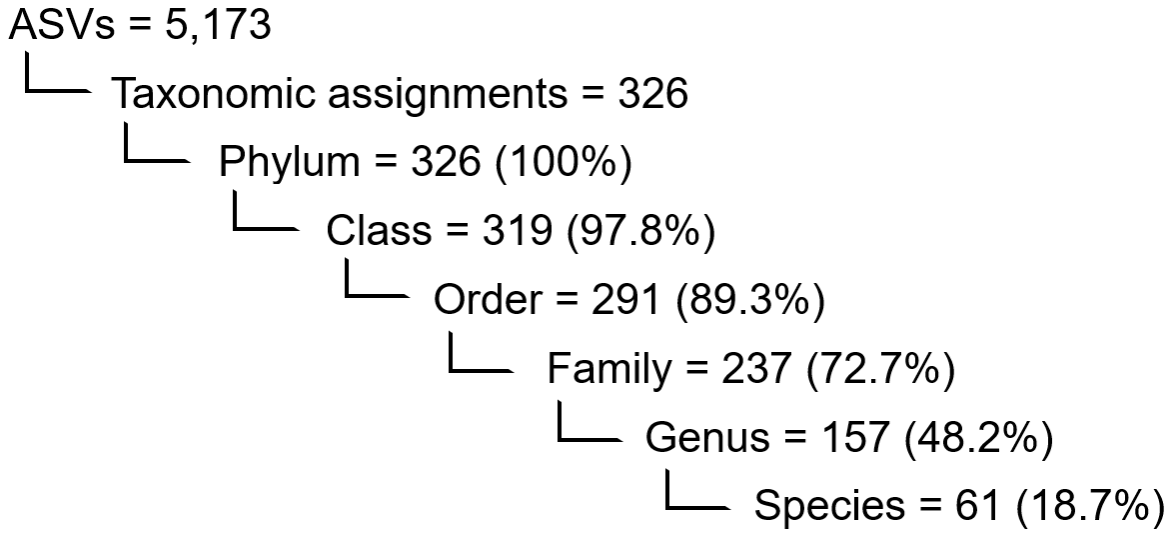
Numerical overview of the detected features in the shell microbiome of apparently healthy lobsters (*N* = 189) and the ASVs within which they were binned. Also indicated are the number and percentage (in brackets) of taxa assigned to each taxonomical level before rarefaction.

During denoising and quality filtering, an average of 9% of the reads were removed and the remaining forward and reverse reads were successfully merged into full denoised sequences. Of the total merged sequence variants, 21% were chimeric and consequently removed by the DADA2 algorithm. A summary of the number and percentage of reads passing the quality filtering steps is given in Supplementary Table S1. After filtering, the PacBio sequencing generated an average of 7,000 reads per sample, of which 5,173 ASVs (amplicon sequence variants) were classified. Overall, 326 taxonomic ranks were assigned to the ASVs, and the percentages assigned to each taxonomic level are shown in Figure 2. The full ASV table is included in the Supplementary Table S2.

### 3.2. Shell microbial community

**Figure 3:**
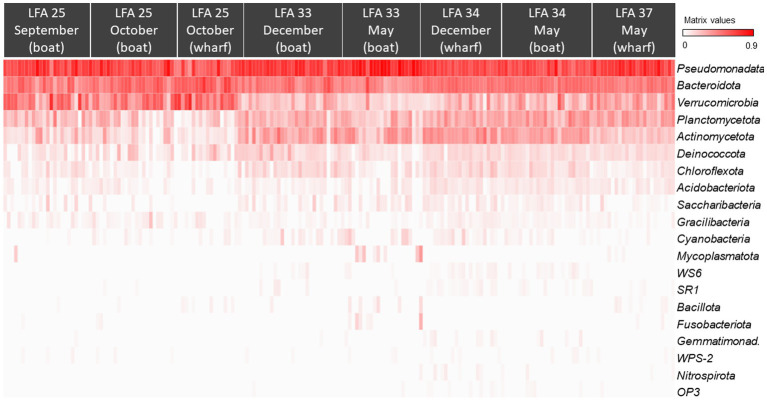
The Hellinger root transformed ASV frequencies per phyla presented in a heat map grouped by sampling location (LFA) and time (month) (*N* = 189). Red colour indicates the highest frequencies of the respective phylum and white the lowest.

All ASVs were at least assigned to the phylum level; the most abundant bacterial phyla were *Pseudomonadata* (formerly: *Proteobacteria*, 46.5%), *Bacteroidota* (formerly: Bacteroidetes, 23.7%), *Verrucomicrobia* (12.4%) and *Actinomycetota* (formerly: *Actinobacteria*, 6.9%). The heatmap in Figure 3 also shows that the shell microbiome of lobsters from LFA 25 (PEI) was richer in *Verrucomicrobia* but lower in *Actinomycetota* and *Planctomycetota* (formerly: *Planctomycetes*). Of the less common phyla, *Bacillota* (formerly: *Firmicutes*), *Mycoplasmatota* (formerly: *Tenericutes*) and *Fusobacteriota* (formerly: *Fusobacteria*) were almost only present in LFA 34 in December (Supplementary Figures S1, S2 show heatmaps of bacterial phyla by sex and moult stage respectively).

**Figure 4:**
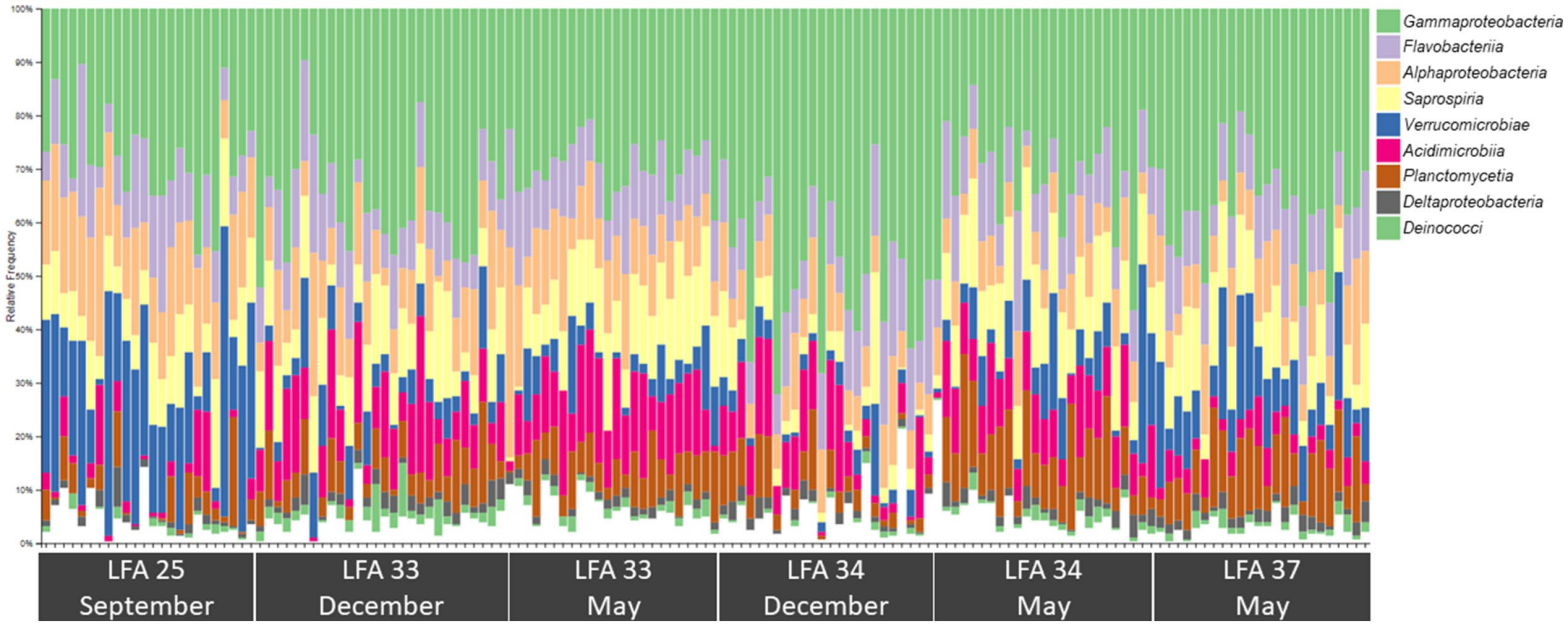
Bar plot showing the relative abundance of the nine most common bacterial classes in the shell microbiome of American lobster (on average these account for just over 96% of the total abundance) *N* = 189.

The most abundant classes in the lobster shell microbiome were *Gammaproteobacteria*, *Saprospiria*, *Verrucomicrobiae*, *Alphaproteobacteria*, *Flavobacteriia*, *Acidimicrobiia*, *Planctomycetia*, *Deltaproteobacteria* and *Deinococci* (Table 2). Their relative abundances are shown in the bar graph in Figure 4 and together they made up 96.7% of the total bacterial community. The relative abundance of some bacterial classes differed between samples from different regions (LFAs) and months. The proportions of *Deinococci*, *Deltaproteobacteria* and *Flavobacteriia* were similar across all sampling regions and times. *Acidimicrobiia* had the lowest relative abundances in LFA 25 sampled in 2022 (1.1%) whereas *Alphaproteobacteria* (16.8%, only in September 2021) and *Verrucomicrobiae* (22.8% in 2021, 28.1% in 2022) had by far the highest proportions there. *Gammaproteobacteria* were highest in LFA 34 in December (51.3%) but lowest in LFA 25 in October 2022 (22.9%). *Planctomycetia* were most abundant in samples from LFA 33, 34, and 37 in May (8.1%–10.7%) and least abundant in LFA 25 (2.0%–4.0%). *Saprospiria* occurred with the highest frequencies in LFA 25 in 2022 (18.8%). Male and female lobsters had similar proportions of most bacterial classes, except *Deinococci* and *Saprospiria* which were more abundant in females and *Acidimicobiia* which was more abundant in males. Berried females (*N* = 7) had less *Gammaproteobacteria* (27.4%) than males (34.1%) and unberried females (33.1%) but more *Planctomycetia*. Female lobsters had a lower frequency of *Acidimicrobiia* (6.3%) than males (8.6%) and berried females (9.0%). Between the moult stages, *Alphaproteobacteria* and *Verrucomicrobiae* were more abundant in postmoult lobsters, and *Acidimicrobiia*, *Gammaproteobacteria* and *Planctomycetia* in intermoult lobsters (Supplementary Tables S3, S4).

**Table 2:** Number of total reads, number of taxa and the relative abundance of the nine most abundant bacterial classes in the shell microbiome of apparently healthy lobsters (*N* = 189).

Class	# Reads	# Taxa	Relative abundance
*Gammaproteobacteria*	399,294	68	33.49
*Saprospiria*	163,663	11	12.14
*Verrucomicrobiae*	155,320	6	12.46
*Alphaproteobacteria*	142,195	64	11.25
*Flavobacteriia*	140,707	40	11.24
*Acidimicrobiia*	98,662	10	6.87
*Planctomycetia*	87,011	7	6.23
*Deltaproteobacteria*	22,353	20	1.62
*Deinococci*	19,382	1	1.42
% of total	96.2%	71.2%	96.7%

The percentage (%) of total refers to the total number of reads and taxa in the dataset.

The most abundant families were *Saprospiraceae* (11.9%), *Verrucomicrobiaceae* (12.4%) *Flavobacteriaceae* (10.0%), and *Thiotrichaceae* (8.7%). Together they made up 42.9% of the shell microbiome on family level. The *Vibrionaceae* family was only represented by six ASVs with a relative abundance of 0.004%. The most abundant genera >1% were *Rubritalea* (12.0%, one ASV), *Cocleimonas* (7.9%, one ASV), *Candidatus* Endobugula (2.2%, one ASV), *Loktanella* (1.6%), *Octadecabacter* (1.8%) and *Maribacter* (1.2%). The genus *Aquimarina* was represented with a relative abundance of 0.4%.

### 3.3. Core microbiome

**Figure 5:**
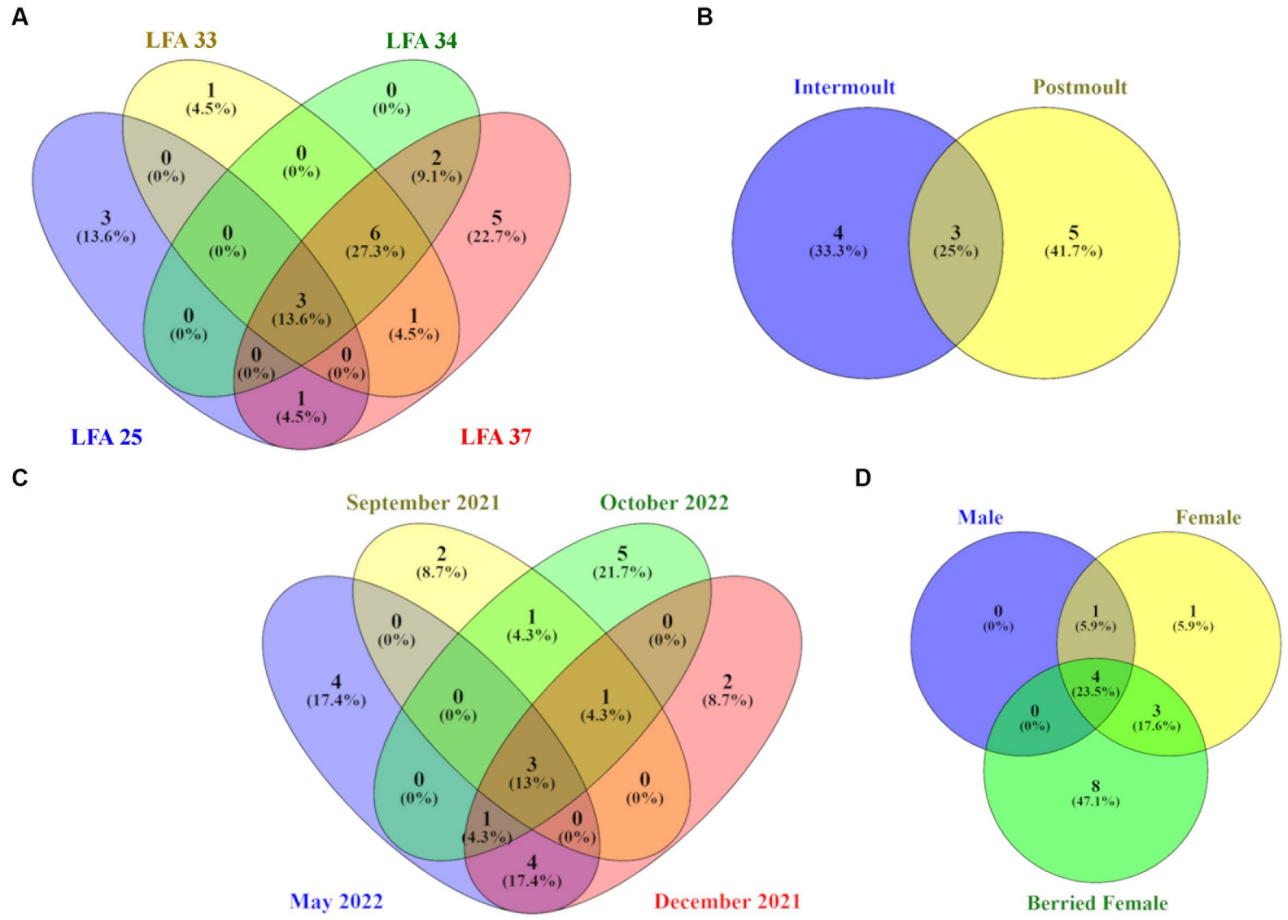
Venn diagrams showing the number and percentage of unique and shared ASVs on the lobster shell microbiome by **(A)** LFA, **(B)** sampling month, **(C)** moult stage and **(D)** sex. Core ASVs were calculated for each category per sampling group respectively, so percentages in the circles refer to the total ASVs identified for each of the four sampling groups.

Venn diagrams show the number and proportion of shared and unique ASVs between sampling groups (Figure 5). Overall, the core microbiome of lobsters was comprised of a total of 24 bacterial taxa and Table 3 summarizes which bacterial taxa differed between sampling groups.

**Table 3:** Table of bacterial taxa (several ASVs may have been assigned to one taxonomic group) comprising the core microbiome and their occurrence by lobster fishing area (LFA), sampling month (5 = May, 9 = September, 10 = October, 12 = December), Sex (B, berried female; F, female; M, male) and moult stage.

Taxa	LFA	Month	Sex	Moult stage
*Cardiobacteriales* (o)	All	All	All	All
*Rubritalea* (g)	All	All	All	All
*Cocleimonas* (g)	All	5, 10, 12	All	All
*Phyllobacteriaceae* (f)	All	All	M, F	Intermoult
*Acidimicrobiales* (f: SC3-41)	33,34,37	5	B	—
*Pirellulaceae* (f)	33,34,37	5, 12	F, B	Intermoult
*Saprospiraceae* (f)	25,33,37	5, 10	B	Postmoult
*Thiohalorhabdales* (o)	33,34,37	All	All	All
*Trueperaceae* (f)	33,34,37	5, 12	F, B	Intermoult
*Maribacter* (g)	34,37	12	F, B	—
*Acidimicrobiales* (f: JdFBGBact)	37	5	B	—
*Alteromonadales* (o)	37	—	B	—
*Candidatus* Endobugula (g)	25	9, 10	—	Postmoult
*Octadecabacter* (g)	37	—	—	—
*Flavobacteriaceae* (f)	—	10	B	-
*Rhodobacteraceae* (f)	—	10	—	Postmoult

NA = bacterial taxon not identified as the core microbiome of this sampling group (in <70% of samples with >0.01% relative abundance). o, order; f, family; g, genus.

On a geographical scale, the LFAs 33, 34 and 37 shared the highest proportion of ASVs (27.3%), while LFA 25 (PEI) and LFA 37 (New Brunswick) samples had the highest proportions of unique ASVs (13.6%, 22.7%). Only 13.6% of ASVs were shared among all LFAs. Seasonally, samples taken in October had the highest number of unique ASVs (21.7%) but the shell microbial community was most similar in May and December with 17.4% of shared features, such as members of the *Pirellulaceae* and the *Trueperaceae* family. Samples from September 2021 and October 2022 were both collected in LFA 25, but only 4.3% of ASVs were shared between the sampling events compared to ASVs that were unique to either sampling event. The shell microbiome of intermoult and postmoult lobsters was not very similar and almost 75% of ASVs were unique to either moult stage. Regarding lobster sex, the highest proportion, with 47.1% of ASVs, was unique to berried females alone, while 23.5% of core ASVs were shared between all three sex groups. Males and females shared only 5.9% of core microbial ASVs.

The core-shell microbiome consisted, for the most part, of the phyla *Bacteroidota*, *Planctomycetota* and *Pseudomonadata*. Of the 16 core taxa identified, only *Cardiobacteriales* and *Rubritalea* were present in all LFAs and sampling months and moult stages. *Cocleimonas* was detected as a core bacterium in all months, sexes, moult stages and months except September. *Thiohalorhabdales* was detected as a core bacterium in all months, sexes, moult stages and LFAs except LFA 25. *Maribacter* and *Trueperaceae* occurred in both berried, and unberried females as a core bacterium.

### 3.4. ESD-associated taxa

Both two most prominent ESD-associated taxa *Aquimarina* (class: *Flavobacteriia*) and *Thalassobius* (class: Alphaproteobacteria) were detected in this study. One species of the genera *Aquimarina, Polaribacter, Pseudoalteromonas and Thalassobius*, two species of the genus *Flavobacterium* and three species of the genera *Sulfitobacter* and *Vibrio* were identified, respectively, in the shell microbiome of Canadian lobsters. *Aquimarina* had the highest mean frequency (8.50, max = 202) and occurred with a prevalence of 47%, whereas *Thalassocius* had the lowest mean frequency (0.05, max = 86) and was found in only 9% of the samples. Of the ESD-associated taxa which have been reported by Chistoserdov et al. (2005, 2012), Bell et al. (2012), Meres et al. (2012) and Ishaq et al. (2023), only *Aquimarina, Polaribacter* and *Sulfitobacter* were detected in sufficient numbers to model explanatory factors for their frequency (Table 4).

**Table 4:** Distribution of taxa that have been previously associated with epizootic shell disease grouped by disease status.

Taxa	ESD	Reads per sample				Non-zero reads	
		Total #Reads	Mean #Reads	SD	Min, max	#Samples	*P* (%)
*Aquimarina* ^a^	H	1,601	8.50	20.3	0.202	89	47.1
	HoD	41	—	—	—	1	100.0
	D	129	—	—	—	1	100.0
*Flavobacterium*	H	95	0.50	2.4	0.18	12	6.3
	HoD	0	—	—	—	0	0.0
	D	0	—	—	—	0	0.0
*Kiloniellaceae (f)*	H	27	0.14	1.5	0.20	3	1.6
	HoD	17	—	—	—	1	100.0
	D	38	—	—	—	1	100.0
*Polaribacter* ^a^	H	282	1.49	4.7	0.45	40	21.2
	HoD	7	—	—	—	1	100.0
	D	0	—	—	—	0	0.0
*Pseudoalteromonas*	H	294	1.56	6.1	0.68	25	13.2
	HoD	0	—	—	—	0	0.0
	D	0	—	—	—	0	0.0
*Sulfitobacter* ^a^	H	1,011	5.35	13.1	0.89	67	35.4
	HoD	0	—	—	—	0	0.0
	D	0	—	—	—	0	0.0
*Thalassobius*	H	10	0.05	0.5	0.6	2	1.1
	HoD	0	—	—	—	0	0.0
	D	0	—	—	—	0	0.0
*Vibrio*	H	163	0.86	6.4	0.86	16	8.5
	HoD	0	—	—	—	0	0.0
	D	0	—	—	—	0	0.0

A total of 189 apparently healthy lobsters (ESD = 0) were included in the occurrence analysis and one lobster was diseased (ESD = 1). P = prevalence (proportion of samples in which these taxa were detected out of 189 samples analyzed). H, healthy shell; HoD, healthy shell on ESD diseased lobster; D, ESD diseased shell; SD, standard deviation.

a Genera included in subsequent negative binomial regression modelling.

**Figure 6:**
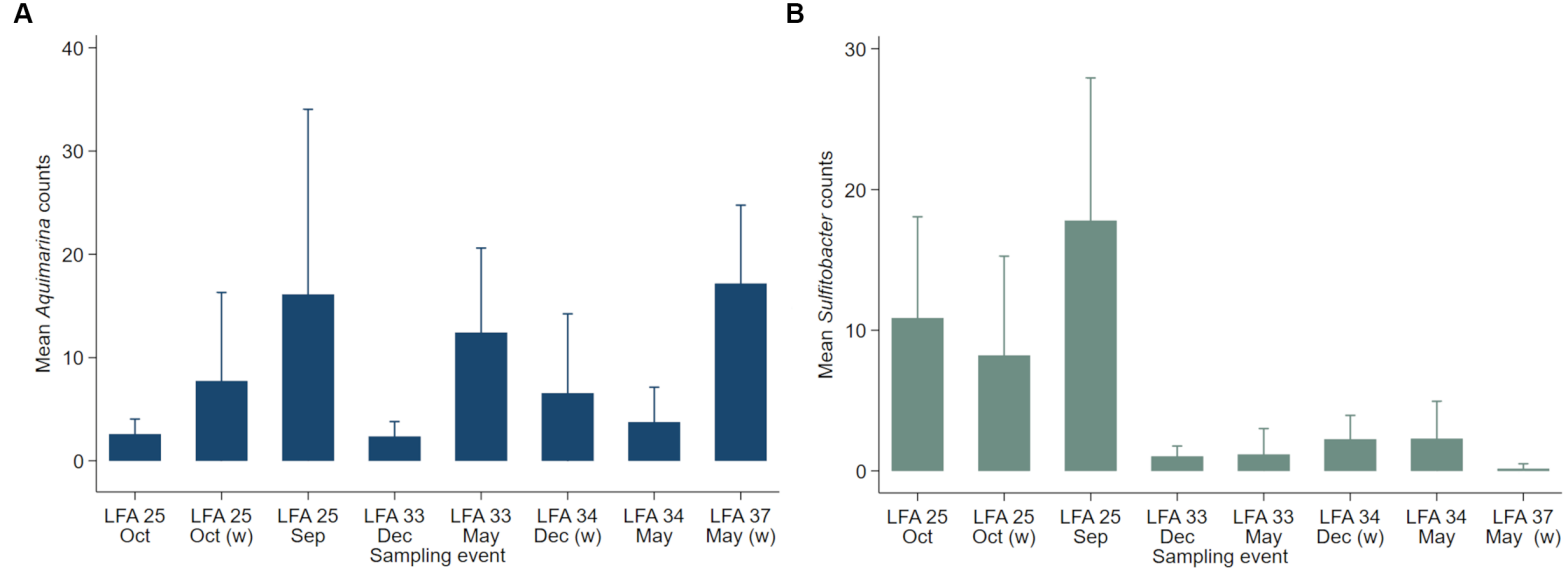
Mean counts (with 95% confidence intervals) of genera **(A)** *Aquimarina* and **(B)** *Sulfitobacter* per LFA and sampling month (*N* = 189). Only the shell microbial data from healthy lobsters were considered for negative binomial analysis.

**Figure 7:**
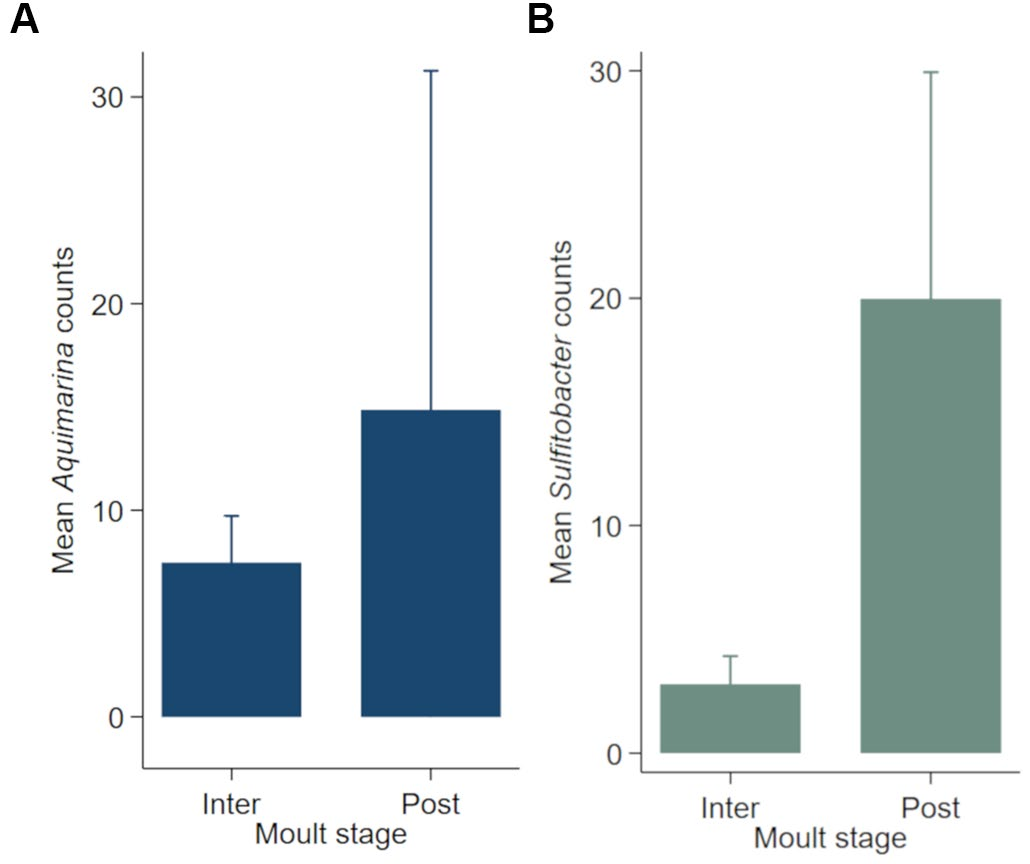
Mean counts (with 95% confidence intervals) of genera **(A)** *Aquimarina* and **(B)** *Sulfitobacter* per moult stage (*N* = 189). Only the shell microbial data from healthy lobsters were considered for negative binomial analysis.

Negative binomial regression models were fitted for all ESD-associated taxa, but only for *Aquimarina, Polaribacter* and *Sulfitobacter* the number of reads differed significantly by the explanatory variables. For *Aquimarina*, LFA and sampling month (e.g., sampling events) as well as the moult stage were significant factors during the forward model building process and were subsequently included in the final NB1 model (sampling event: *p* = 0.000; moult stage: *p* = 0.029, Wald-test). This indicated that the frequency of *Aquimarina* depended on the sampling area and sampling season and whether lobsters were in inter- or postmoult. The predicted counts for *Aquimarina* were highest in LFA 37 (New Brunswick) with an estimated 26.4 reads per sample, and more than double in postmoult lobsters (16.3 estimated reads per sample) compared to intermoult lobsters (7.7 reads per sample). For *Sulfitobacter*, LFA and sampling month (*p* = 0.000, Wald test)—combined as sampling event—and moult stage (*p* = 0.005, Wald test) were significant predictors in the final NB1 model. The predicted *Sulfitobacter* counts were highest in LFA 25 with an estimated 10.7 reads per sample (September 2021) and in postmoult lobsters with 9.0 reads as compared to intermoult lobsters (4.0). For *Polaribacter*, lobster sex was the only significant factor in the final NB1 model, and berried females had the highest estimated reads per sample with 3.37. The negative binomial regression model parameters for all analyzed taxa are shown in Table 5 and the mean *Sulfitobacter* and *Aquimarina* counts per sampling events and moult stage are shown in Figures 6, 7. The results for the rarefied dataset within the framework of the sensitivity analysis are presented in the Supplementary Tables S5, S6 and Supplementary Figure S3. Briefly, the overall patterns of the estimates from the negative binomial regression models did not differ much between the rarefied and non-rarefied dataset. *Aquimarina* counts were highest in LFA 37 and *Sulfitobacter* counts were highest in LFA 25 in post moult lobsters in both approaches. However, when using the rarefied dataset, *Polaribacter* occurrence did not differ significantly by lobster sex, and *Aquimarina* occurrence did not differ significantly by moult stage. This information loss could be due to the implemented rarefaction step, which led us to present the raw data as our main results.

**Table 5:** The factor variables and respective outputs of the negative binomial regression models fitted to the frequency of each bacterial genus.

Genus	Model variables		Coefficient	SE	*p*-value
*Aquimarina*	Sampling event	LFA 25 (October)	Reference category		
		LFA 25 (October, w)	−0.052	0.473	0.000
		LFA 25 (September)	0.071	0.448	
		LFA 33 (December)	0.024	0.436	
		LFA 33 (May)	0.519	0.436	
		LFA 34 (Dec)	0.037	0.453	
		LFA 34 (May)	−0.253	0.495	
		LFA 37 (May)	1.508	0.378	
	Moult stage	Intermoult	Reference category		
		Postmoult	0.758	0.340	0.029
		Intercept	1.621	0.351	
		Delta	3.681	0.209	
*Polaribacter*	Sex	Female	Reference category		
		Male	−0.909	0.331	0.011
		Berried female	0.336	0.608	
		Intercept	0.819	0.289	
		Delta	2.831	0.323	
*Sulfitobacter*	Sampling event	LFA 25 (October)	Reference category		
		LFA 25 (October, w)	−0.171	0.383	0.000
		LFA 25 (September)	0.145	0.36	
		LFA 33 (December)	−1.109	0.435	
		LFA 33 (May)	−1.901	0.633	
		LFA 34 (December)	−0.907	0.433	
		LFA 34 (May)	−1.595	0.562	
		LFA 37 (May)	−3.107	1.033	
	Moult stage	Intermoult	Reference category		
		Postmoult	0.822	0.294	0.005
		Intercept	2.062	0.287	
		Delta	3.321	0.237	

*N* = 189, SE, standard error.

The Blast assignment identified 12 different strains of *Aquimarina* (Table 6) and *A. algiphila A. amphilecti*, *A. macrocephali*, *A. muelleri* and *A. pacifica* were identified to species level. *Aquimarina* strain AQ103 and *Aquimarina algiphila* had the highest prevalences with 18.0% and 15.9%, respectively.

**Table 6:** *Aquimarina* species and strains identified by a Blast analysis, including the accession numbers of the closest match in the database as well as the number of reads and ASV the respective taxa had and in how many samples they were detected.

*Aquimarina* species^a^	Strain	Percent identity	Query cover	Accession length	Accession number	# Reads	# Samples	# ASVs
*A. algiphila*	PMIC1E1A	100.0%	85.0%	1,238	MW740084.1	326	30	1
*A. amphilecti*	92V	97.9%	91.0%	1,327	NR_126246.1	29	7	3
*A. macrocephali*	JAMB N27	97.7%	100.0%	1,467	NR_112971.1	302	10	2
*A. muelleri*	HAL205	95.8%	100.0%	1,516	MT406596.1	8	3	1
*A. pacifica*	SW150	96.5%	100.0%	1,483	NR_133696.1	33	5	2
*Aquimarina* sp.	3C	97.7%	95.0%	1,381	MT484143.1	153	14	2
*Aquimarina* sp.	AQ103	98.5%	94.0%	1,369	HE818265.1	297	34	4
*Aquimarina* sp.	AQ132	98.7%	96.0%	1,389	HE818115.1	63	3	1
*Aquimarina* sp.	AQ77	99.8%	94.0%	1,368	HE818243.1	24	5	1
*Aquimarina* sp.	CP19	98.3%	100.0%	1,510	MH061266.1	82	9	2
*Aquimarina* sp.	TP207	96.0%	93.0%	1,373	KT900248.1	435	28	4

a Closest described relative based on Blast analysis (http://blast.ncbi.nlm.nih.gov/Blast.cgi).

## 4. Discussion

This study collected and analyzed shell microbial samples from over 180 lobsters from Nova Scotia, New Brunswick and Prince Edward Island; within the most important lobster fishing areas in Canada (LFA 33 and 34). As almost all animals were free of shell disease and sampled from areas with reportedly low to no ESD prevalence, these results provide new insights into the shell microbial community of apparently healthy lobsters. We detected that sampling time and sampling region, as well as to some extent moult stage, rather than lobster sex or size, influence the shell microbiome; that potential pathogens, as well as potential beneficial bacterial taxa, are present in the core microbiome; and that the frequency of taxa previously associated with ESD depends on geographic and seasonal factors. Due to the large sample size as well as the standardized sampling and processing protocols, the presented microbiome data can also serve as a relevant baseline reference for future studies.

### 4.1. Boat vs. wharf sampling

Although the sampling protocol for this study was standardized as much as possible, differences in sample collection—either from the boat or from the wharf—persisted. Hence, a direct comparison of the effect of these methods on the resulting shell microbiome was implemented in LFA 25. There were differences in the relative abundance of some bacterial taxa, for example, the class *Gammaproteobacteria* had lower relative abundance in samples taken from the wharf. Still, these observations were in line with the overall trend of shell microbial samples from LFA 25 having a lower representation of this class than other regions. Furthermore, the heatmaps and relative abundance bar plots do not show obvious differences in boat and wharf samples from the same sampling event. Therefore, we assume that both sampling methods yield similar outputs and that results are comparable across this study.

### 4.2. The overall shell microbial community

With around 5,000 ASVs detected, this study shows a similar microbial richness as others that have analyzed shell microbiota in American lobster (Zha et al., 2019; Schaubeck et al., 2023) and other crustaceans (Zha et al., 2019; Kraemer et al., 2020) in the wild. In some studies of lab-reared lobsters, up to 18,000 taxonomic units (similar to ASVs) were detected (Feinmann et al., 2017). The higher richness in shell samples from tank-based compared to wild animals is potentially linked to higher bacterial load in tank environments. While the opposite was observed in wild and captive eagle rays (Clavere-Graciette et al., 2022) and tank-acclimated horseshoe crabs (Friel et al., 2020), there is limited data on the microbial difference between wild and tank-based crustaceans.

The most common phyla detected on shells of apparently healthy lobsters are comparable to what has been observed on other crustaceans. *Actinomycetota*, *Bacteroidota*, *Planctomycetota*, and *Pseudomonadota* were previously found on healthy American lobsters (Chistoserdov et al., 2012; Feinmann et al., 2017). In addition to the aforementioned phyla, Feinmann et al. (2017) also detected *Deferribacteraceae* in apparently healthy lab-reared American lobsters, which were not present in our dataset. *Bacillota*, *Fusobacteriota* and *Mycoplasmatota* were less abundant on lobsters in this study, but they are common marine taxa and have been previously recorded on shells of lobsters and brown crabs (Payne et al., 2008; Bell et al., 2012; Meres et al., 2012; Quinn et al., 2013; Bergen et al., 2022). Bell et al. (2012) showed that, among others, *Bacillota*, *Fusobacteriota* and *Mycoplasmatota* were over-represented in healthy compared to shell-diseased American lobsters. Here, we saw a higher frequency of these three taxa in LFA 34 in December compared to the same region in spring or to LFA 33 in December. In addition to disease state (not analyzed in this study), it appears that geographical and seasonal factors play a role in the distribution of these phyla.

In this study, we confirmed previous observations that the most common bacterial classes on lobster carapaces are *Acidimicrobiia*, *Alphaproteobacteria*, *Flavobacteriia*, *Gammaproteobacteria*, *Saprospiria* and *Verrucomicrobiae* (Chistoserdov et al., 2012; Meres et al., 2012; Quinn et al., 2013; Ishaq et al., 2023). Some of these classes showed differential distributions between sampling sites and times. While there are studies on how the bacterial community differs by shell disease state on lobsters, significantly less is known about the effect of spatiotemporal and host factors on bacterial community. For example, *Verrucomicrobiae* had higher relative abundance in healthy compared to shell-diseased spiny lobsters in New Zealand (Zha et al., 2019) and *Alphaproteobacteria* levels were higher in American lobsters without ESD (Meres et al., 2012). We observed that *Verrucomicrobiae* and *Alphaproteobacteria* abundances were highest in LFA 25 in September and in postmoult lobsters. At the same time, LFA 25 had the highest proportion of postmoult lobsters, so it is difficult to discern whether a higher abundance of these taxa can be attributed to location or to the moult stage. A recent study found that on rock crabs (*Cancer irrotatus*) carapaces, *Verrucomicrobiae* also had the highest abundance in the Northumberland Straight (close proximity to LFA 25) compared to other regions in Atlantic Canada, which also points towards geographical effects on the lobster shell microbial community (Koepper et al., 2023). *Saprospiria* has only been isolated from lobster shells once (Andrews, 2018, unpublished thesis), but colonized the gut and hemolymph of spiny lobsters (Ooi et al., 2017, 2019). In the present study, *Saprospiria* was the least common in LFA 34 in December but had almost 10% higher abundance during May of the following year. Members of the *Saprospiria* class are sensitive to environmental factors such as temperature and CO_2_ in benthic communities (Raulf et al., 2015; Hicks et al., 2018). If conditions are less favourable for this class during winter, this could explain the seasonal difference in their relative abundance in the lobster shell microbiome.

*Planctomycetia*, which were the least abundant in LFA 25 (both years), are a dominant bacterial class in coastal sediments (Feinmann et al., 2017; Hicks et al., 2018). They have also been found to be dominant in healthy shrimp and absent in shell-diseased brown crabs (Chen et al., 2017; Bergen et al., 2022). Lastly, members of the *Acidimicrobiia* were previously detected in healthy (not shell-diseased) lobsters (Chistoserdov et al., 2012; Quinn et al., 2013; Feinmann et al., 2017; Zha et al., 2019) and brown crabs (Bergen et al., 2022).

### 4.3. The core microbial composition

Of the 16 bacterial taxa identified as core microbes on lobster shells, all have previously been detected on crustacean carapaces except *Acidimicrobiales* (family: SC3-41, JdFBGBact), *Alteromonadales* and *Trueperaceae*. The families SC3-41 and JdFBGBact of the *Acidimicrobiales* and *Pirellulaceae* have been detected before in water samples from salmon farms and on the seaweed *Sargassum muticum* as an epibiont (Rud et al., 2017; Serebryakova et al., 2018). While members of the *Trueperaceae* have not yet been found on lobster carapaces, Ooi et al. (2019) detected *Trueperaceae* in the hemolymph of lab-reared spiny lobsters (*Panulirus ornatus*) where the taxa occurred at higher frequencies in control compared to thermally stressed hosts.

Most other core bacterial taxa found in our study have been previously associated with shell disease in American lobsters and other crustaceans. Two of the four taxa that were positively linked to shell disease by previous research and present here as core bacteria were shared between most sampling groups: *Cardiobacteriales* and *Thiohalorhabdales*. Members of the order *Cardiobacteriales* have only been found in shell disease lesions and never on healthy carapaces of American lobsters albeit with overall low frequency (Quinn et al., 2013). Here, this taxon was observed in the core microbiome in all sampling groups (LFAs, months, sexes and moult stages). Similarly, the order *Thiohalorhabdales* has been associated with tail fan necrosis lesions in spiny lobsters previously (Zha et al., 2019). While the genus *Maribacter* occurs both on lobster shells free of ESD and on ESD lesions, Chistoserdov et al. (2012) and Schaubeck et al. (2023) reported a higher frequency of this genus in ESD lesions (Quinn et al., 2012, 2013). Here, *Maribacter* is a core bacterium on lobster shells sampled from LFA 33 and 37 and in berried and unberried females. *Flavobacteriaceae* are a ubiquitous family in crustacean shell microbiomes, but they contain genera that have previously been associated with ESD and other shell diseases (*Aquimarina*) and, as such, *Flavobacteriaceae* abundance increases in shell disease lesions (Chistoserdov et al., 2005; Zha et al., 2019). Here, members of *Flavobacteriaceae* were core bacteria only in October and in berried females.

Three taxa previously associated with non-shell diseased crustaceans, and present here as core bacteria, were shared between all sampling groups: *Cocleimonas*, *Saprospiraceae* and *Rubritalea*. *Cocleimonas* previously occurred in both shell-diseased and healthy brown crabs (Bergen et al., 2022), but only colonized non-ESD American lobster (Quinn et al., 2013; Feinmann et al., 2017). *Saprospiraceae* were most commonly identified from non-ESD lobsters and from cuticles unaffected by shell disease (Feinmann et al., 2017; Andrews, 2018; Zha et al., 2019). The genus *Rubritalea* is a common epibiont on marine animals and has been associated with healthy (non-ESD) lobster shells in previous research (Feinmann et al., 2017; Ishaq et al., 2023). This genus produces carotenoids, which may have antimicrobial functions and interestingly, ESD-diseased lobsters have fewer carotenoids on their shells (Prince and Bayer, 2005). Therefore, *Rubritalea* could be a beneficial shell colonizer for their hosts. Furthermore, two known marine symbionts were part of the core microbiome only in one LFA respectively: *Candidatus* Endobugula in LFA 25 and *Octadecabacter* in LFA 37. Mathew and Lopanik (2014) detected the symbiotic relationship between members of the genus *Candidatus* Endobugula and its bryozoan host, but in lobsters, this microbe was majorly isolated from shells that were transitioning into shell disease (Feinmann et al., 2017). *Octadecabacter* is a dominant subcuticular symbiont in brittle stars (Morrow et al., 2018), but in American lobsters, this genus has been detected with an increased presence in shell-diseased lobsters and in ESD lesions (Chistoserdov et al., 2012; Quinn et al., 2012; Andrews, 2018; Zha et al., 2019).

Another core microbial taxon of the family *Phyllobacteriaceae* was shared by all sampling regions and times in this study. *Phyllobacteriaceae* were both isolated from healthy cuticles and ESD lesions in American lobsters, and an ubiquitous distribution of this family is coherent (Quinn et al., 2012; Feinmann et al., 2017). The *Rhodobacteraceae* family is ubiquitous on lobster carapaces and has been detected in healthy lobsters as well as in shell disease lesions (Chistoserdov et al., 2012; Quinn et al., 2013; Feinmann et al., 2017). In shell diseased brown crabs, Bergen et al. (2022) detected a shift toward this bacterial family. Here, *Rhodobacteraceae* were part of the core microbiome only in October (LFA 25) in postmoult lobsters. This could indicate their role as early colonizers of the new shell, as they commonly dominate marine biofilms (Elifantz et al., 2013).

### 4.4. Differential frequency of ESD-associated taxa

The data of this study provided us with a unique opportunity to demonstrate that the distribution of shell microbial taxa previously associated with ESD is influenced by location and sampling time as well as by host factors.

The genera considered to be associated with ESD *Aquimarina, Polaribacter, Pseudoalteromonas, Sulfitobacter, Thalassobius* and *Vibrio* have been found in the shell microbiome of American lobsters from Atlantic Canada before, but not the genera *Flavobacterium* and *Kiloniella* (Quinn et al., 2013). In our samples of apparently healthy American lobsters, all the above-mentioned genera were present. However, some occurred with a frequency too low to evaluate factors associated with their distribution. With a high number of sampled lobsters and several sampling events, both taxa most commonly associated with ESD were detected, although with low relative frequencies (*Aquimarina*: 0.1%, *Thalassobius*: 0.001%). In a previous analysis of 75 lobsters (ESD diseased and healthy) sampled from the northern and southern Gulf of Maine (US), *Thalassobius* did not occur in the shell microbiome (Ishaq et al., 2023). Quinn et al. (2013) have reported that the distribution of ESD-associated taxa in Atlantic Canada differs from Southern New England populations. This may be because these disease-associated taxa would be less common in ESD-free lobster populations or in regions of the Gulf of Maine and Gulf of St. Lawrence compared to Southern New England.

Although only one diseased lobster was collected in this study, ESD disease state affected the frequency of the two ESD-associated taxa when this lobster was included in the analyses. If not, the time and location of the sampling event, as well as lobster moult stage significantly affected the distribution of *Aquimarina* and *Sulfitobacter* in our models. The occurrence of *Aquimarina* was highest in LFA 37, a fishing area close in proximity to LFA 35 in the Bay of Fundy where ESD prevalence in Atlantic Canada is anecdotally the highest (Petritchenko, personal communication, 2023). A seasonal difference in *Aquimarina* counts was seen in LFA 33, where they were higher in May than December. ESD has been associated with higher temperatures and the ESD peak in the Gulf of Maine is observed during May and June (Reardon et al., 2018). *Aquimarina* counts were also significantly higher in postmoult lobsters compared to intermoult lobsters. Postmoult lobsters have younger shells, however, ESD has been associated with individuals with older shells (Groner et al., 2018). Lower *Aquimarina* counts in older shells, which seem to be a risk factor for ESD, was an unexpected result and may emphasize the opportunistic nature of this genus on healthy lobsters. For *Sulfitobacter* our model indicated that both the LFA and the sampling month, as well as moult stages were significantly associated with its frequency in the samples. Predicted *Sulfitobacter* counts were highest in LFA 25 and in postmoult lobsters. As most of the postmoult lobsters were sampled in LFA 25, it is likely that this taxon is more common in this fishing region, where more postmoult animals happened to be sampled. Large, female lobsters are reportedly more susceptible to ESD as they usually moult only every 2 years (Glenn and Pugh, 2006; Castro and Somers, 2012; Hoenig et al., 2017). Lobsters with a longer intermoult phase have a longer exposure to changes in their shell microbiome and potentially to pathogens. The negative binomial models developed here indicate that lobster sex and size do not predict the frequency of ESD-associated taxa *Aquimarina* and *Sulfitobacter.* While it has been shown that males, females and berried females react differently to environmental stressors and prefer different habitats according to their size (Koepper et al., 2021), this may not have a large influence on their shell microbial community in healthy individuals. The genus *Polaribacter* was significantly more abundant on shells of berried female lobsters, although only a small number (*N* = 7) of this population group was sampled. While berried females are reportedly more susceptible to ESD (Groner et al., 2018), our results should be interpreted with caution as our study only considered healthy lobsters.

Due to the rarity of *Aquimarina*, this genus is considered to belong to the microbial rare biosphere. In this study, *Aquimarina* was less rare (relative abundance = 0.4%) than in a recent survey where the relative abundance was mostly below 0.1% over several marine environments (Silva et al., 2022). In contrast, on healthy lobster shells sampled from Long Island Sound, a region with historically high rates of ESD, the relative abundance of *Aquimarina* was around 4% (Schaubeck et al., 2023). The blast analysis showed that several *Aquimarina* species and strains are present on healthy American lobster shells. *A. muelleri* and *A. macrocephali* have previously been detected on ESD lesions in Atlantic Canada and on black spot lesions of brown crabs, although *A. macrocephali* was also isolated from healthy crabs in the same study (Quinn et al., 2013; Bergen et al., 2022). Although not yet isolated from ESD lesions, one of the most prevalent *Aquimarina* species detected here, *A. algiphila* (prevalence = 15.9%) can degrade chitin (Nedashkovskaya et al., 2018) and is closely related to *A. pacifica*. The strain that has most commonly been associated with ESD lesions, *A.* “*homaria*” strain I32.4 was not found in this study (Chistoserdov et al., 2012; Ranson et al., 2018). Accordingly, this strain seems to be rare in healthy lobsters in Atlantic Canada. However, other strains may also have the capability to act opportunistically when encountering a suitable host, and successively could be involved in ESD progression. So far, no work has been carried out on the distribution of these taxa in healthy lobsters in Atlantic Canada and no consistent ESD monitoring is currently being implemented in the region. This makes it difficult to determine whether differences in the frequency of these taxa stem from geographic variation alone or if a higher frequency could be linked to lobsters being more likely to develop ESD.

## 5. Limitations

As sampling for this study took place on commercial lobster fishing vessels, sampling times were restricted to the respective fishing seasons. This is the reason not all regions could be sampled at the same time, potentially introducing seasonal differences in the microbial community that are difficult to account for. For example, the shell microbial community in LFA 25 in September showed differences in microbial composition when compared to samples taken in other regions during May. However, it was not possible to sample LFA 25 in May for better spatial comparison as the fishing season there only lasts from mid-August until mid-October. While we analyzed all sampling events by LFA and months separately to account for this limitation, future studies could profit from a more balanced sampling design. Retrieving samples from commercial lobster boats also led to targeting more intermoult lobsters compared to freshly moulted (postmoult) animals, because fishing usually takes place well after the moulting season. Only in LFA 25, where samples were taken not long after the lobster moult, more postmoult lobsters were represented in the samples. If postmoult lobsters have a distinct shell microbial community, when compared to intermoult lobsters, this could have caused bias, as it is difficult to determine whether the microbial differences stem from a geographic influence or because more postmoult lobsters were present in the samples. Additionally, not many berried female lobsters were sampled, potentially because they are usually not targeted by the fishery. Having roughly equal numbers of males, females and berried females would enable a better microbial comparison to be made between those groups, given that berried females have also been shown to be more susceptible to shell disease. Furthermore, no seawater controls or blanks could be sequenced as these samples contained too little DNA for PacBio sequencing. This inhibited the control for any potential contaminating ASVs in the shell samples. Nevertheless, the consistency in sample collection, processing and analysis makes this a valuable dataset for a first description of healthy lobster microbiomes and for crustacean microbiome studies in the future.

## 6. Conclusion

This comprehensive analysis of the shell microbial community of American lobster highlights differences in relative abundances of the most common bacterial phyla and classes. These differences are more pronounced when comparing samples from different LFAs and at different times of the year, than by the host factors (such as lobster sex, size or moult condition). The marine epibiotic community is highly variable and depends on environmental factors such as temperature and salinity. Epibionts can also provide the host with important metabolic products (Wahl et al., 2012). LFA 25 stands out with the highest abundance of *Verrucomicrobiae* and the lowest abundance of *Acidimicrobiia* and *Planctomycetia*, and while *Planctomycetia* has been associated with healthy crustaceans, it is unknown in what way this may affect the local lobster populations, if at all. Of 16 core microbial taxa, only two occurred in all LFAs and sampling months, and four occurred in both males and (berried) females and in intermoult and postmoult lobsters. This indicates a certain degree of variation in the distribution of the core microbiome and could mean that bacterial taxa play a distinct role in the microbial community depending on where the host is located as well as the time of year. Some potential pathogens (e.g., *Cardiobacteriales* and *Thiohalorhabdales*) and beneficial bacteria (e.g., *Rubritalea*) were shared among regions and sampling times. However, a potential symbiont *Candidatus* Endobugula was found only in the core microbiome in shell samples from LFA 25, whereas *Flavobacteriaceae*, which are enriched on shell-diseased lobsters, were only identified as core epibionts in samples from October (LFA 25) and berried females. Furthermore, it was shown that the occurrence of ESD-associated taxa, *Aquimarina* in particular, depends on the geographic area, sampling time and the lobster moult stage. The unique composition of the core microbiome, together with distinct distributions of the ESD-associated taxa, highlights the differences in the microbial community on healthy lobster shells from Atlantic Canada. It has yet to be determined whether this is concomitant with lobsters being more predisposed to shell disease in areas where ESD-associated taxa frequency is highest or where the core microbiome features certain potential pathogens. In this study, we provided a detailed overview of the *Aquimarina* species and strains detected in the dataset, which will be a useful tool for ESD research in Canadian water and beyond. For future studies, we encourage a holistic approach in shell disease monitoring, combined with microbiome assessment of healthy and diseased lobsters, to elucidate the risk factors for this important disease.

## Data availability statement

The datasets presented in this study can be found in online repositories. The names of the repository/repositories and accession number(s) can be found at: https://www.ncbi.nlm.nih.gov/bioproject/PRJNA1026623/.

## Ethics statement

The manuscript presents research on animals that do not require ethical approval for their study.

## Author contributions

SK, KFC, KKT, and CWR designed the research. SK collected the data and wrote the manuscript. SK and HS analyzed the data. SK, KFC, JTM, CWR, HS, and KKT revised and edited the manuscript. All authors contributed to the article and approved the submitted version.
