# Supplementary material for: Long-read sequencing reveals the shell microbiome of apparently healthy American lobsters Homarus americanus from Atlantic Canada
Source: Front Microbiol. 2023 Nov 6;14:1245818. doi: 10.3389/fmicb.2023.1245818 (PMC10658194; doi:10.3389/fmicb.2023.1245818)
Supplement: Supplementary file 1 [file Data_Sheet_1.docx]

**Supplementary Figures**

**Long-read sequencing reveals the shell microbiome of apparently healthy American lobsters *Homarus americanus* from Atlantic Canada**

Koepper S.^1^, Clark K.F.^2^, McClure J^1^., Revie C.W.^3^, Stryhn H.^1^, Thakur K.K.^1^

^1^Department of Health Management, Atlantic Veterinary College, University of Prince Edward Island, Charlottetown C1A 4P3 PE, Canada

^2^Department of Animal Sciences and Aquaculture, Faculty of Agriculture, Dalhousie University, Truro, NS B2N 5E3, Canada

^3^Department of Computer and Information Sciences, University of Strathclyde, Glasgow G1 1XQ, UK

**Table S1:** Dada2 denoising statistics per sample.

| **Sample-ID** | **Input** | **Filtered** | **Percentage of input passed filter** | **Denoised** | **Non-chimeric** | **Percentage of input non-chimeric** |
| --- | --- | --- | --- | --- | --- | --- |
| Sample-110 | 11519 | 10567 | 91.74 | 10194 | 10194 | 88.5 |
| Sample-111 | 18407 | 16854 | 91.56 | 16203 | 16203 | 88.03 |
| Sample-112 | 24581 | 22166 | 90.18 | 20041 | 20039 | 81.52 |
| Sample-113 | 731 | 675 | 92.34 | 599 | 599 | 81.94 |
| Sample-114 | 11000 | 10038 | 91.25 | 9482 | 9482 | 86.2 |
| Sample-115 | 11720 | 10732 | 91.57 | 10187 | 10171 | 86.78 |
| Sample-117 | 9244 | 8431 | 91.21 | 7740 | 7740 | 83.73 |
| Sample-118 | 462 | 418 | 90.48 | 342 | 342 | 74.03 |
| Sample-119 | 16539 | 15112 | 91.37 | 14363 | 14363 | 86.84 |
| Sample-120 | 6021 | 5491 | 91.2 | 5085 | 5085 | 84.45 |
| Sample-121 | 5484 | 5009 | 91.34 | 4738 | 4738 | 86.4 |
| Sample-122 | 456 | 408 | 89.47 | 293 | 293 | 64.25 |
| Sample-123 | 2975 | 2760 | 92.77 | 2538 | 2538 | 85.31 |
| Sample-124 | 8917 | 8168 | 91.6 | 7515 | 7515 | 84.28 |
| Sample-125 | 11073 | 9908 | 89.48 | 8085 | 8018 | 72.41 |
| Sample-126 | 5664 | 5190 | 91.63 | 4712 | 4712 | 83.19 |
| Sample-127 | 11728 | 10719 | 91.4 | 10028 | 10028 | 85.5 |
| Sample-128 | 3417 | 3085 | 90.28 | 2837 | 2837 | 83.03 |
| Sample-129 | 12849 | 11750 | 91.45 | 11190 | 11190 | 87.09 |
| Sample-130 | 12691 | 11598 | 91.39 | 10860 | 10807 | 85.15 |
| Sample-131 | 16340 | 14957 | 91.54 | 13471 | 13433 | 82.21 |
| Sample-132 | 9350 | 8574 | 91.7 | 7927 | 7927 | 84.78 |
| Sample-133 | 10209 | 9303 | 91.13 | 8902 | 8902 | 87.2 |
| Sample-134 | 929 | 859 | 92.47 | 763 | 761 | 81.92 |
| Sample-137 | 5335 | 4846 | 90.83 | 4675 | 4665 | 87.44 |
| Sample-138 | 13366 | 12127 | 90.73 | 11332 | 11332 | 84.78 |
| Sample-139 | 12681 | 11515 | 90.81 | 10786 | 10774 | 84.96 |
| Sample-140 | 15380 | 14047 | 91.33 | 13073 | 13058 | 84.9 |
| Sample-141 | 9360 | 8554 | 91.39 | 7788 | 7788 | 83.21 |
| Sample-142 | 3187 | 2922 | 91.68 | 2693 | 2693 | 84.5 |
| Sample-143 | 4266 | 3922 | 91.94 | 3638 | 3638 | 85.28 |
| Sample-144 | 1851 | 1675 | 90.49 | 1498 | 1498 | 80.93 |
| Sample-145 | 11881 | 10906 | 91.79 | 10224 | 10224 | 86.05 |
| Sample-146 | 2189 | 2006 | 91.64 | 1809 | 1809 | 82.64 |
| Sample-147 | 8746 | 8051 | 92.05 | 7539 | 7515 | 85.92 |
| Sample-148 | 13802 | 12573 | 91.1 | 11324 | 11324 | 82.05 |
| Sample-149 | 11007 | 9829 | 89.3 | 8647 | 8606 | 78.19 |
| Sample-150 | 12171 | 10999 | 90.37 | 10022 | 10012 | 82.26 |
| Sample-151 | 6291 | 5718 | 90.89 | 5241 | 5241 | 83.31 |
| Sample-152 | 4321 | 3933 | 91.02 | 3612 | 3612 | 83.59 |
| Sample-153 | 4098 | 3781 | 92.26 | 3507 | 3485 | 85.04 |
| Sample-154 | 14625 | 13344 | 91.24 | 12562 | 12529 | 85.67 |
| Sample-155 | 13346 | 12088 | 90.57 | 10657 | 10657 | 79.85 |
| Sample-156 | 9654 | 8827 | 91.43 | 8254 | 8254 | 85.5 |
| Sample-157 | 3869 | 3525 | 91.11 | 3232 | 3232 | 83.54 |
| Sample-158 | 11328 | 10204 | 90.08 | 9076 | 9042 | 79.82 |
| Sample-159 | 4765 | 4315 | 90.56 | 3934 | 3934 | 82.56 |
| Sample-160 | 12180 | 11051 | 90.73 | 10343 | 10333 | 84.84 |
| Sample-161 | 14877 | 13527 | 90.93 | 12428 | 12428 | 83.54 |
| Sample-162 | 12904 | 11691 | 90.6 | 10614 | 10614 | 82.25 |
| Sample-163 | 14233 | 12925 | 90.81 | 12033 | 12033 | 84.54 |
| Sample-164 | 11290 | 10274 | 91 | 9691 | 9691 | 85.84 |
| Sample-167 | 5859 | 5205 | 88.84 | 3800 | 3767 | 64.29 |
| Sample-168 | 5431 | 4946 | 91.07 | 4506 | 4506 | 82.97 |
| Sample-169 | 13927 | 12579 | 90.32 | 11668 | 11657 | 83.7 |
| Sample-170 | 10375 | 9347 | 90.09 | 7686 | 7678 | 74 |
| Sample-171 | 2075 | 1862 | 89.73 | 1638 | 1638 | 78.94 |
| Sample-172 | 13328 | 12120 | 90.94 | 11169 | 11169 | 83.8 |
| Sample-173 | 4594 | 4137 | 90.05 | 3182 | 3182 | 69.26 |
| Sample-174 | 752 | 690 | 91.76 | 504 | 504 | 67.02 |
| Sample-176 | 4534 | 4145 | 91.42 | 3508 | 3508 | 77.37 |
| Sample-178 | 8129 | 7451 | 91.66 | 6440 | 6440 | 79.22 |
| Sample-179 | 5507 | 4925 | 89.43 | 3731 | 3721 | 67.57 |
| Sample-180 | 1050 | 958 | 91.24 | 729 | 729 | 69.43 |
| Sample-181 | 12322 | 11140 | 90.41 | 9418 | 9396 | 76.25 |
| Sample-182 | 500 | 466 | 93.2 | 387 | 387 | 77.4 |
| Sample-183 | 1586 | 1433 | 90.35 | 1177 | 1166 | 73.52 |
| Sample-184 | 4943 | 4531 | 91.66 | 3832 | 3819 | 77.26 |
| Sample-185 | 893 | 807 | 90.37 | 686 | 686 | 76.82 |
| Sample-186 | 14348 | 13093 | 91.25 | 12180 | 12144 | 84.64 |
| Sample-187 | 6125 | 5595 | 91.35 | 5161 | 5144 | 83.98 |
| Sample-188 | 6178 | 5637 | 91.24 | 5042 | 5042 | 81.61 |
| Sample-189 | 736 | 674 | 91.58 | 554 | 554 | 75.27 |
| Sample-190 | 1494 | 1363 | 91.23 | 1066 | 1066 | 71.35 |
| Sample-191 | 2000 | 1815 | 90.75 | 1676 | 1676 | 83.8 |
| Sample-192 | 549 | 514 | 93.62 | 406 | 406 | 73.95 |
| Sample-193 | 6600 | 6083 | 92.17 | 5570 | 5531 | 83.8 |
| Sample-196 | 20305 | 18287 | 90.06 | 14644 | 14559 | 71.7 |
| Sample-197 | 15480 | 13913 | 89.88 | 10538 | 10421 | 67.32 |
| Sample-198 | 38324 | 34277 | 89.44 | 25168 | 24981 | 65.18 |
| Sample-199 | 39857 | 35524 | 89.13 | 27509 | 27234 | 68.33 |
| Sample-200 | 2802 | 2575 | 91.9 | 2237 | 2237 | 79.84 |
| Sample-201 | 23723 | 21278 | 89.69 | 15788 | 15580 | 65.67 |
| Sample-202 | 22081 | 19706 | 89.24 | 14438 | 14398 | 65.21 |
| Sample-203 | 23521 | 21051 | 89.5 | 15941 | 15905 | 67.62 |
| Sample-204 | 11155 | 10042 | 90.02 | 8074 | 8031 | 71.99 |
| Sample-206 | 14679 | 13181 | 89.79 | 11245 | 11112 | 75.7 |
| Sample-207 | 12360 | 11042 | 89.34 | 9164 | 9100 | 73.62 |
| Sample-208 | 14080 | 12645 | 89.81 | 9540 | 9538 | 67.74 |
| Sample-209 | 12052 | 10782 | 89.46 | 8310 | 8263 | 68.56 |
| Sample-210 | 16108 | 14411 | 89.46 | 10757 | 10716 | 66.53 |
| Sample-211 | 1577 | 1430 | 90.68 | 1039 | 1039 | 65.88 |
| Sample-212 | 18484 | 16640 | 90.02 | 13462 | 13411 | 72.55 |
| Sample-213 | 9514 | 8600 | 90.39 | 6936 | 6936 | 72.9 |
| Sample-214 | 12196 | 10868 | 89.11 | 8488 | 8440 | 69.2 |
| Sample-215 | 10882 | 9822 | 90.26 | 7268 | 7234 | 66.48 |
| Sample-216 | 13857 | 12473 | 90.01 | 9193 | 9190 | 66.32 |
| Sample-217 | 15062 | 13554 | 89.99 | 10982 | 10945 | 72.67 |
| Sample-218 | 13846 | 12450 | 89.92 | 9936 | 9798 | 70.76 |
| Sample-219 | 11518 | 10318 | 89.58 | 8112 | 8092 | 70.26 |
| Sample-222 | 9890 | 8839 | 89.37 | 7093 | 7093 | 71.72 |
| Sample-223 | 9035 | 8102 | 89.67 | 6184 | 6179 | 68.39 |
| Sample-224 | 9296 | 8365 | 89.98 | 6805 | 6800 | 73.15 |
| Sample-225 | 10652 | 9614 | 90.26 | 7197 | 7191 | 67.51 |
| Sample-226 | 3605 | 3286 | 91.15 | 2615 | 2615 | 72.54 |
| Sample-227 | 9222 | 8294 | 89.94 | 6577 | 6577 | 71.32 |
| Sample-228 | 8251 | 7378 | 89.42 | 5648 | 5629 | 68.22 |
| Sample-229 | 10863 | 9743 | 89.69 | 7421 | 7400 | 68.12 |
| Sample-230 | 11584 | 10311 | 89.01 | 7698 | 7414 | 64 |
| Sample-231 | 10292 | 9235 | 89.73 | 7249 | 7249 | 70.43 |
| Sample-232 | 10254 | 9164 | 89.37 | 7344 | 7316 | 71.35 |
| Sample-233 | 9961 | 9005 | 90.4 | 6956 | 6953 | 69.8 |
| Sample-234 | 12093 | 10933 | 90.41 | 8579 | 8563 | 70.81 |
| Sample-236 | 10409 | 9358 | 89.9 | 7283 | 7165 | 68.83 |
| Sample-237 | 9331 | 8377 | 89.78 | 7160 | 7160 | 76.73 |
| Sample-238 | 13185 | 11844 | 89.83 | 8533 | 8527 | 64.67 |
| Sample-239 | 8680 | 7794 | 89.79 | 5778 | 5759 | 66.35 |
| Sample-240 | 12665 | 11451 | 90.41 | 8445 | 8441 | 66.65 |
| Sample-241 | 13780 | 12408 | 90.04 | 9778 | 9689 | 70.31 |
| Sample-242 | 10658 | 9558 | 89.68 | 7159 | 7040 | 66.05 |
| Sample-243 | 10114 | 9055 | 89.53 | 6238 | 6233 | 61.63 |
| Sample-244 | 7163 | 6447 | 90 | 4976 | 4872 | 68.02 |
| Sample-245 | 11567 | 10559 | 91.29 | 8735 | 8724 | 75.42 |
| Sample-246 | 11690 | 10587 | 90.56 | 8257 | 8226 | 70.37 |
| Sample-250 | 8473 | 7754 | 91.51 | 6282 | 6219 | 73.4 |
| Sample-251 | 7887 | 7096 | 89.97 | 5238 | 5191 | 65.82 |
| Sample-252 | 10003 | 9068 | 90.65 | 7278 | 7247 | 72.45 |
| Sample-253 | 8551 | 7855 | 91.86 | 7351 | 7351 | 85.97 |
| Sample-254 | 12875 | 11582 | 89.96 | 9366 | 9316 | 72.36 |
| Sample-255 | 9060 | 8142 | 89.87 | 6516 | 6486 | 71.59 |
| Sample-256 | 9207 | 8291 | 90.05 | 6906 | 6882 | 74.75 |
| Sample-257 | 11286 | 10245 | 90.78 | 8223 | 8206 | 72.71 |
| Sample-258 | 4221 | 3862 | 91.49 | 3282 | 3282 | 77.75 |
| Sample-259 | 13320 | 12013 | 90.19 | 9267 | 9179 | 68.91 |
| Sample-260 | 13874 | 12560 | 90.53 | 9931 | 9917 | 71.48 |
| Sample-261 | 15594 | 13976 | 89.62 | 10863 | 10795 | 69.23 |
| Sample-262 | 9124 | 8208 | 89.96 | 6034 | 6034 | 66.13 |
| Sample-263 | 1938 | 1783 | 92 | 1531 | 1531 | 79 |
| Sample-264 | 6654 | 6023 | 90.52 | 4670 | 4666 | 70.12 |
| Sample-266 | 10738 | 9669 | 90.04 | 7531 | 7505 | 69.89 |
| Sample-267 | 12040 | 10655 | 88.5 | 7919 | 7673 | 63.73 |
| Sample-268 | 9176 | 8221 | 89.59 | 6640 | 6617 | 72.11 |
| Sample-269 | 12177 | 10980 | 90.17 | 8885 | 8848 | 72.66 |
| Sample-270 | 12867 | 11563 | 89.87 | 9363 | 9211 | 71.59 |
| Sample-271 | 12350 | 11209 | 90.76 | 9336 | 9288 | 75.21 |
| Sample-272 | 12092 | 10804 | 89.35 | 8834 | 8776 | 72.58 |
| Sample-273 | 6688 | 6040 | 90.31 | 5496 | 5496 | 82.18 |
| Sample-274 | 11013 | 9924 | 90.11 | 7951 | 7949 | 72.18 |
| Sample-277 | 1436 | 1348 | 93.87 | 1172 | 1172 | 81.62 |
| Sample-278 | 7743 | 7167 | 92.56 | 5864 | 5861 | 75.69 |
| Sample-279 | 3394 | 3172 | 93.46 | 2910 | 2910 | 85.74 |
| Sample-280 | 7087 | 6629 | 93.54 | 6237 | 6237 | 88.01 |
| Sample-281 | 678 | 630 | 92.92 | 533 | 533 | 78.61 |
| Sample-282 | 7582 | 7024 | 92.64 | 6513 | 6513 | 85.9 |
| Sample-283 | 5154 | 4810 | 93.33 | 4627 | 4627 | 89.77 |
| Sample-284 | 5185 | 4827 | 93.1 | 4342 | 4312 | 83.16 |
| Sample-285 | 3619 | 3376 | 93.29 | 2958 | 2958 | 81.74 |
| Sample-286 | 9774 | 9091 | 93.01 | 8242 | 8242 | 84.33 |
| Sample-287 | 9684 | 8939 | 92.31 | 7854 | 7854 | 81.1 |
| Sample-288 | 1796 | 1680 | 93.54 | 1484 | 1484 | 82.63 |
| Sample-289 | 5429 | 5024 | 92.54 | 4638 | 4638 | 85.43 |
| Sample-290 | 6536 | 6021 | 92.12 | 5211 | 5211 | 79.73 |
| Sample-291 | 5904 | 5497 | 93.11 | 4998 | 4998 | 84.65 |
| Sample-292 | 5974 | 5556 | 93 | 5037 | 4994 | 83.6 |
| Sample-293 | 10041 | 9321 | 92.83 | 8512 | 8481 | 84.46 |
| Sample-294 | 9825 | 9239 | 94.04 | 8772 | 8755 | 89.11 |
| Sample-295 | 8851 | 8278 | 93.53 | 7697 | 7697 | 86.96 |
| Sample-296 | 10413 | 9778 | 93.9 | 9254 | 9254 | 88.87 |
| Sample-298 | 10634 | 9908 | 93.17 | 9284 | 9284 | 87.3 |
| Sample-299 | 3206 | 2998 | 93.51 | 2828 | 2828 | 88.21 |
| Sample-300 | 8442 | 7897 | 93.54 | 7198 | 7181 | 85.06 |
| Sample-302 | 2570 | 2436 | 94.79 | 2191 | 2191 | 85.25 |
| Sample-305 | 2089 | 1991 | 95.31 | 1799 | 1799 | 86.12 |
| Sample-306 | 597 | 568 | 95.14 | 467 | 467 | 78.22 |
| Sample-307 | 1103 | 1031 | 93.47 | 869 | 869 | 78.79 |
| Sample-308 | 1721 | 1611 | 93.61 | 1385 | 1385 | 80.48 |
| Sample-309 | 1268 | 1198 | 94.48 | 1094 | 1094 | 86.28 |
| Sample-310 | 9754 | 9110 | 93.4 | 8387 | 8385 | 85.96 |
| Sample-311 | 15682 | 14625 | 93.26 | 13379 | 13370 | 85.26 |
| Sample-312 | 5498 | 5155 | 93.76 | 4808 | 4808 | 87.45 |
| Sample-313 | 9940 | 9259 | 93.15 | 8668 | 8668 | 87.2 |
| Sample-314 | 8412 | 7791 | 92.62 | 6761 | 6761 | 80.37 |
| Sample-315 | 4507 | 4238 | 94.03 | 3908 | 3908 | 86.71 |
| Sample-316 | 8552 | 7985 | 93.37 | 7416 | 7399 | 86.52 |
| Sample-317 | 8953 | 8374 | 93.53 | 7693 | 7691 | 85.9 |
| Sample-318 | 8035 | 7469 | 92.96 | 6724 | 6724 | 83.68 |
| Sample-319 | 8223 | 7676 | 93.35 | 7031 | 7031 | 85.5 |
| Sample-320 | 10943 | 10173 | 92.96 | 8892 | 8843 | 80.81 |
| Sample-321 | 2545 | 2381 | 93.56 | 2234 | 2234 | 87.78 |
| Sample-322 | 9209 | 8594 | 93.32 | 7448 | 7415 | 80.52 |
| Sample-323 | 4471 | 4200 | 93.94 | 3905 | 3892 | 87.05 |
| Average | 9164.99 | 8322.71 | 91.23 | 7059.88 | 7036.92 | 77.96 |

**Table S2:** ASV-frequency table

PROVIDED IN SEPARATE FILE

**Table S3:** Relative abundance of the nine most abundant taxa by LFA and sampling month.

|  | **REGION AND SEASON** | | | | | | | | | | | | | | | |
| --- | --- | --- | --- | --- | --- | --- | --- | --- | --- | --- | --- | --- | --- | --- | --- | --- |
|  | LFA 25 | | | | | | LFA 33 | | | | LFA 34 | | | | LFA 37 | |
|  | Sep (2021) | | Oct (2022) | | Oct (2022) | | May (2022) | | Dec (2021) | | May (2022) | | Dec (2021) | | May (2022) | |
| Class | Boat | | Boat | | Wharf | | Boat | | Boat | | Boat | | Wharf | | Wharf | |
| *Acidimicrobiia* | 3.37 | 0.55 | 1.84 | 0.41 | 1.05 | 0.26 | 13.34 | 1.90 | 11.53 | 1.82 | 10.66 | 1.60 | 9.92 | 1.58 | 3.79 | 0.63 |
| *Alphaproteobacteria* | 17.03 | 0.93 | 12.61 | 0.73 | 10.80 | 0.65 | 10.32 | 0.36 | 11.31 | 0.54 | 7.51 | 0.26 | 9.26 | 0.40 | 9.29 | 0.42 |
| *Deinococci* | 0.70 | - | 1.87 | - | 1.63 | - | 1.74 | - | 2.20 | - | 1.33 | - | 0.88 | - | 1.37 | - |
| *Deltaproteobacteria* | 1.24 | 0.14 | 1.75 | 0.15 | 1.40 | 0.12 | 1.46 | 0.13 | 2.41 | 0.36 | 1.67 | 0.14 | 1.76 | 0.26 | 2.16 | 0.23 |
| *Flavobacteriia* | 11.00 | 0.95 | 10.31 | 1.01 | 13.83 | 1.60 | 10.72 | 1.06 | 10.06 | 1.04 | 10.75 | 1.02 | 11.88 | 1.20 | 11.29 | 0.92 |
| *Gammaproteobacteria* | 26.86 | 1.33 | 28.74 | 1.59 | 21.01 | 1.07 | 29.46 | 1.42 | 35.69 | 1.66 | 29.77 | 1.45 | 42.09 | 1.91 | 36.83 | 1.86 |
| *Planctomycetia* | 4.32 | 1.48 | 3.85 | 1.37 | 1.75 | 0.60 | 7.88 | 2.27 | 6.62 | 1.90 | 10.87 | 3.41 | 6.61 | 1.91 | 9.68 | 3.42 |
| *Saprospiria* | 10.35 | 3.00 | 16.87 | 4.85 | 19.94 | 5.86 | 13.06 | 3.80 | 10.83 | 3.09 | 14.46 | 4.21 | 8.13 | 2.38 | 11.11 | 3.16 |
| *Verrucomicrobiae* | 21.06 | 8.51 | 20.73 | 8.42 | 27.53 | 11.22 | 6.20 | 2.13 | 5.18 | 1.93 | 8.70 | 3.11 | 4.32 | 1.53 | 11.87 | 4.70 |
| Percent of total (%) | 95.92 |  | 98.56 |  | 98.95 |  | 94.19 |  | 95.81 |  | 95.73 |  | 94.84 |  | 97.40 |  |

**Table S4**: Relative abundance of the nine most abundant taxa by lobster sex and moult stage.

|  | **SEX** | | | | | | **MOULT STAGE** | | | |
| --- | --- | --- | --- | --- | --- | --- | --- | --- | --- | --- |
|  | Male | | Female | | Berried | | Intermoult | | Postmoult | |
|  |  |  |  |  |  |  |  |  |  |  |
| Class |  |  |  |  |  |  |  |  |  |  |
| *Acidimicrobiia* | 8.60 | 1.27 | 6.32 | 0.98 | 8.95 | 1.35 | 8.47 | 1.28 | 2.82 | 0.42 |
| *Alphaproteobacteria* | 11.31 | 0.45 | 10.91 | 0.47 | 10.60 | 0.49 | 10.41 | 0.41 | 15.79 | 0.83 |
| *Deinococci* | 1.16 | - | 1.98 | - | 2.03 | - | 1.67 | - | 0.51 | - |
| *Deltaproteobacteria* | 1.88 | 0.22 | 1.57 | 0.15 | 1.71 | 0.15 | 1.86 | 0.19 | 1.01 | 0.12 |
| *Flavobacteriia* | 10.99 | 1.05 | 11.09 | 1.06 | 10.61 | 1.03 | 10.79 | 1.04 | 12.50 | 1.14 |
| *Gammaproteobacteria* | 32.11 | 1.49 | 30.37 | 1.39 | 27.60 | 1.31 | 32.09 | 1.50 | 25.80 | 1.21 |
| *Planctomycetia* | 6.51 | 2.02 | 6.95 | 2.22 | 9.79 | 3.01 | 7.43 | 2.33 | 2.78 | 0.90 |
| *Saprospiria* | 11.78 | 3.41 | 14.07 | 4.06 | 15.37 | 5.18 | 12.94 | 3.74 | 11.99 | 3.50 |
| *Verrucomicrobiae* | 11.32 | 4.41 | 13.61 | 5.33 | 9.85 | 3.75 | 10.49 | 4.04 | 23.01 | 9.34 |
| Percent of total (%) | 95.67 |  | 96.86 |  | 96.52 |  | 96.16 |  | 96.22 |  |

**Table S5**: Distribution of taxa that have been previously associated with epizootic shell disease grouped by disease status for the rarefied dataset (400 reads per sample). A total of 185 apparently healthy lobsters (ESD = 0) were included in the occurrence analysis and one lobster was diseased (ESD = 1). P = prevalence (proportion of samples in which these taxa were detected out of 185 samples analyzed), *genera included in subsequent negative binomial regression modelling. H = healthy shell, HoD = healthy shell on ESD diseased lobster, D = ESD diseased shell, SD = standard deviation.

|  |  | **Reads per sample** | | | | **Non-zero reads** | |
| --- | --- | --- | --- | --- | --- | --- | --- |
| **Taxa** | **ESD** | **Total #reads** | **Mean #reads** | **SD** | **min, max** | **#samples** | **P (%)** |
| *Aquimarina ** | H | 40 | 0.42 | 1.36 | 0,11 | 40 | 21.6 |
|  | HoD | 4 | - | - |  | 1 | 100.0 |
|  | D | 17 | - | - |  | 1 | 100.0 |
| Kiloniellaceae (f) | H | 1 | 0.05 | 0.01 | 0,1 | 1 | 0.5 |
|  | HoD | 2 | - | - |  | 1 | 100.0 |
|  | D | 5 | - | - |  | 5 | 100.0 |
| *Polaribacter* | H | 31 | 0.17 | 0.79 | 0.9 | 14 | 7.6 |
|  | HoD | 0 | - | - |  | 0 | 0.0 |
|  | D | 0 | - | - |  | 0 | 0.0 |
| *Pseudoalteromonas* | H | 40 | 0.21 | 1.06 | 0.8 | 13 | 7.0 |
|  | HoD | 0 | - | - |  | 0 | 0.0 |
|  | D | 0 | - | - |  | 0 | 0.0 |
| *Sulfitobacter** | H | 76 | 0.41 | 1.22 | 0,7 | 35 | 18.9 |
|  | HoD | 0 | - | - |  | 0 | 0.0 |
|  | D | 0 | - | - |  | 0 | 0.0 |
| *Vibrio* | H | 19 | 0.10 | 1.23 | 0,15 | 5 | 2.7 |
|  | HoD | 0 | - | - |  | 0 | 0.0 |
|  | D | 0 | - | - |  | 0 | 0.0 |
|  |  |  |  |  |  |  |  |

**Table S6**: The factor variables and respective outputs of the negative binomial regression models fitted to the frequency of each bacterial genus for the rarefied dataset. N = 185, P = prevalence, SE = standard error.

| **Genus** | **Model variables** | | **Coefficient** | **SE** | **P-value** |
| --- | --- | --- | --- | --- | --- |
| *Aquimarina* | Sampling event | LFA 25 (Oct) | Reference category | | |
|  |  | LFA 25 (Oct, w) | -0.399 | 0.863 | 0.017 |
|  |  | LFA 25 (Sep) | -0.143 | 0.761 |  |
|  |  | LFA 33 (Dec) | -0.886 | 0.865 |  |
|  |  | LFA 33 (May) | 0.730 | 0.621 |  |
|  |  | LFA 34 (Dec) | 0.580 | 0.642 |  |
|  |  | LFA 34 (May) | 0.111 | 0.703 |  |
|  |  | LFA 37 (May) | 1.310 | 0.571 |  |
|  |  | Intercept | -1.249 | 0.512 |  |
|  |  | Delta | 1.553 | 0.541 |  |
| *Sulfitobacter* | Sampling event | LFA 25 (Oct) | Reference category | | |
|  |  | LFA 25 (Oct, w) | -0.146 | 0.443 | 0.002 |
|  |  | LFA 25 (Sep) | -0.339 | 0.417 |  |
|  |  | LFA 33 (Dec) | -1.831 | 0.777 |  |
|  |  | LFA 33 (May) | -2.219 | 1.056 |  |
|  |  | LFA 34 (Dec) | -1.631 | 0.767 |  |
|  |  | LFA 34 (May) | -1.517 | 0.782 |  |
|  |  | LFA 37 (May) | -16.443 | 1200.765 |  |
|  | Moult stage | Intermoult | Reference category | | |
|  |  | Postmoult | 1.436 | 0.358 | 0.000 |
|  |  | Intercept | -0.520 | 0.340 |  |
|  |  | Delta | 1.112 | 0.477 |  |

**Fig. S1:** The Hellinger transformed ASV frequencies per phyla presented in a heat map grouped by lobster sex (males, females and berried females). Red colour indicates the highest frequencies of the respective phylum and white the lowest.

**Fig. S2:** The Hellinger transformed ASV frequencies per phyla presented in a heat map grouped by lobster moult stage (intermoult and postmoult). Red colour indicates the highest frequencies of the respective phylum and white the lowest.

**Fig. S3**: Mean counts (with 95% confidence intervals) of genera (**A**) *Aquimarina* and (**B**) *Sulfitobacter* per LFA and sampling month for the rarefied dataset (N = 185). Only the shell microbial data from healthy lobsters were considered for negative binomial analysis.

B

A
